# Correlations between mitochondrial DNA haplogroup D5 and chronic hepatitis B virus infection in Yunnan, China

**DOI:** 10.1038/s41598-018-19184-6

**Published:** 2018-01-17

**Authors:** Xiao Li, Tai-Cheng Zhou, Chang-Hui Wu, Li-Lin Tao, Rui Bi, Li-Jun Chen, De-Yao Deng, Chang Liu, Newton O. Otecko, Yang Tang, Xin Lai, Liang Zhang, Jia Wei

**Affiliations:** 1 Central Lab, Liver Disease Research Center, the Second People’s Hospital of Yunnan Province, Kunming, 650203 Yunnan China; 2 Key Laboratory of Animal Models and Human Disease Mechanisms of the Chinese Academy of Sciences & Yunnan Province, Kunming Institute of Zoology, Kunming, 650223 Yunnan China; 3 Clinical Laboratory of the Second People’s Hospital of Yunnan Province, Kunming, 650203 Yunnan China; 4 State Key Laboratory of Genetic Resources and Evolution & Yunnan Laboratory of Molecular Biology of Domestic Animals, Kunming Institute of Zoology, Chinese Academy of Sciences, Kunming, 650223 China; 5 Kunming College of Life Science, University of Chinese Academy of Sciences, Kunming, 650204 China; 6 The First Affiliated Hospital of Kunming Medical University, Kunming, Yunnan Province 650000 China

## Abstract

Mitochondrial abnormality is frequently reported in individuals with hepatitis B virus (HBV) infection, but the associated hosts’ mitochondrial genetic factors remain obscure. We hypothesized that mitochondria may affect host susceptibility to HBV infection. In this study, we aimed to detect the association between chronic HBV infection and mitochondrial DNA in Chinese from Yunnan, Southwest China. A total of 272 individuals with chronic HBV infection (CHB), 310 who had never been infected by HBV (healthy controls, HC) and 278 with a trace of HBV infection (spontaneously recovered, SR) were analysed for mtDNA sequence variations and classified into respective haplogroups. Haplogroup frequencies were compared between HBV infected patients, HCs and SRs. Haplogroup D5 presented a higher frequency in CHBs than in HCs (*P* = 0.017, OR = 2.87, 95% confidence interval [CI] = (1.21–6.81)) and SRs (*P* = 0.049, OR = 2.90, 95% CI = 1.01–8.35). The network of haplogroup D5 revealed a distinct distribution pattern between CHBs and non-CHBs. A trend of higher viral load among CHBs with haplogroup D5 was observed. Our results indicate the risk potential of mtDNA haplogroup D5 in chronic HBV infection in Yunnan, China.

## Introduction

Hepatitis B is one of the world’s leading public health problems caused by hepatitis B virus (HBV) infection. An estimated 257 million people are infected with HBV (defined as hepatitis B surface antigen positive) and an appreciable amount of people die every year due to complications of chronic HBV infection, including cirrhosis and liver cancer. Hepatitis B prevalence is highest in sub-Saharan Africa and East Asia, where between 5–10% of the adult population is chronically infected^1^ (WHO, Hepatitis B, Fact sheet, http://www.who.int/mediacentre/factsheets/fs204/en/). The course of HBV infection is influenced by age at infection, HBV replication level and host immune status^2,3^. Recently, several genome-wide association studies (GWASs) and a considerable amount of studies have uncovered genetic factors that may confer susceptibility to hepatitis B^4–12^, suggesting that host genetic background is most likely to take part in chronic HBV infection. However, the scope of the potential genetic predispositions to HBV infection remains murky, and their role is yet to be determined at functional level.

Mitochondrion is the power plant of all cells and body cells contain variable number of mitochondrial DNA (mtDNA) copies. The displacement loop (D-loop), also known as mtDNA control region, is necessary for replication and transcription of the mitochondrial genome^13^. Mitochondria modulate many cell events including energy metabolism, calcium signaling, cell apoptosis and production of free radicals^14–16^. Free radicals can damage mtDNA, resulting in more free radical generation^17^. Alteration in mitochondrial genetic factors or functions may lead to many kinds of diseases such as psychiatric disorder, neural system dysfunction, myocardial diseases and infectious diseases^18–20^. Whether mitochondrial genetic factor may affect chronic HBV infection has not been investigated.

The HBV genome encodes four partially overlapped open reading frames (ORF), One of the ORFs, the ‘*x*’ gene, encodes a 17 KDa regulatory protein HBx consisting of 154 amino acids^21^. HBx is necessary for viral infection as it has been reported to likely play important roles during the establishment of infection, host cell apoptosis, hepatocarcinogenesis and other virus-host cell interactions^22^. HBx is mainly localised in the cytoplasm^23^, often co-localising with mitochondria^24^. In 1995, Zhang *et al*. demonstrated that HBx can form complex with human mitochondrial HSP60 and HSP70, signifying mitochondria as potential targets of HBx^22^. HBx can increase mitochondrial calcium uptake and promote store-operated calcium entry (SOCE) to sustain higher cytosolic calcium, which stimulate HBV replication^25^. Experimental blockage of mitochondrial permeability transition pore caused a decrease in both mitochondrial and cytosolic calcium, and a concomitant inhibition of HBV replication^25,26^. Taken together, mitochondria may play important role in HBV infection which requires careful investigation.

In this study, we hypothesized that the process of HBV infection may be strongly dependent on host energy production, which is anchored on the mitochondrial genomic infrastructure, hence the link with disease susceptibility. To test this hypothesis, we sequenced the mitochondrial D-loop region of a general population fromYunnan Province, Southwest China. The study population comprised individuals with chronic HBV infection (CHB), spontaneously recovered (SR) subjects after HBV infection and healthy controls (HC) who had never been infected with HBV. We compared their mtDNA haplogroup frequencies and clinical characters. Our results showed that mtDNA haplogroup D5 is associated with chronic HBV infection. Whether haplogroup D5 confers risk to impaired liver function after chronic HBV infection needs further investigation.

## Results

### Clinical and demographic characteristics of participants

The clinical and demographic characteristics of the 860 individuals enrolled in this study (272 CHBs, 278 SRs and 310 HCs) were summarized in Table 1. The variables analysed include gender, age, serum markers of HBV, serum levels of alanine transaminase (ALT), aspartate aminotransferase (AST), total bilirubin (TBIL), direct bilirubin (DBIL), total protein (TP) and albumin (ALB). There were more female members in SR group than in CHB and HC groups (*P* < 0.0001) and the male SR subjects were much older (*P* < 0.0001) in our hospital-based sample enrollment. There was a significant difference in serum ALT, AST and DBIL levels between CHBs and the other two groups. Gender and age structure did not differ significantly between CHBs and HCs. Among the clinical parameters of liver function, AST level was higher in SRs than in HCs (*P* = 0.024) (Table 1), possibly influenced by population stratification on behalf of clinical outcomes. No significant difference was observed in other parameters between these two groups.

**Table 1:** Clinical and demographic characteristics of participants.

	CHB	SR	HC	CHB vs. SR	CHB vs. HC	SR vs. HC
Gender (M/F)	164/108	104/174	196/114	*P* < 0.0001	*P* = 0.468	*P* < 0.0001
Age of M (Y), (mean ± SD)	42.7 ± 13.5	52.5 ± 16.7	42.9 ± 11.9	*P* < 0.0001	*P* = 0.869	*P* < 0.0001
Age of F (Y), (mean ± SD)	41.4 ± 14.4	43.4 ± 16.2	43.6 ± 11.5	*P* = 0.289	*P* = 0.211	*P* = 0.929
HBsAg	All+	All−	All−			
Anti-HBs, n (%)	All−	All+	215 (69.35)			
HBeAg, n (%)	62 (22.79)	All−	All−			
Anti-HBc	All+	All+	All−			
ALT (IU/L)	42.42 ± 62.03	25.55 ± 20.57	28.27 ± 18.84	*P* < 0.0001	*P* = 0.0002	*P* = 0.104
AST (IU/L)	33.11 ± 32.36	25.79 ± 19.60	23.01 ± 8.06	*P* = 0.008	*P* < 0.0001	*P* = 0.024
TBIL (IU/L)	15.58 ± 15.88	13.49 ± 9.98	13.77 ± 5.48	*P* = 0.075	*P* = 0.061	*P* = 0.671
DBIL (IU/L)	5.59 ± 5.80	4.58 ± 6.31	4.28 ± 1.84	*P* = 0.058	*P* = 0.0002	*P* = 0.428

M, male; F, female; Y, years; SD, standard deviation; CHB, chronic HBV infected individuals; SR, spontaneously recovered individuals with history of HBV infection; HC, healthy controls without HBV infection or history of HBV infection; ALT, alanine transaminase; AST, aspartate aminotransferase; TBIL, total bilirubin; DBIL, directed bilirubin.

The differences of clinical characteristics between each two groups were calculated by chi-square test and student’s t test (unpaired).

### Association between haplogroup distribution and chronic HBV infection

**Figure 1:**
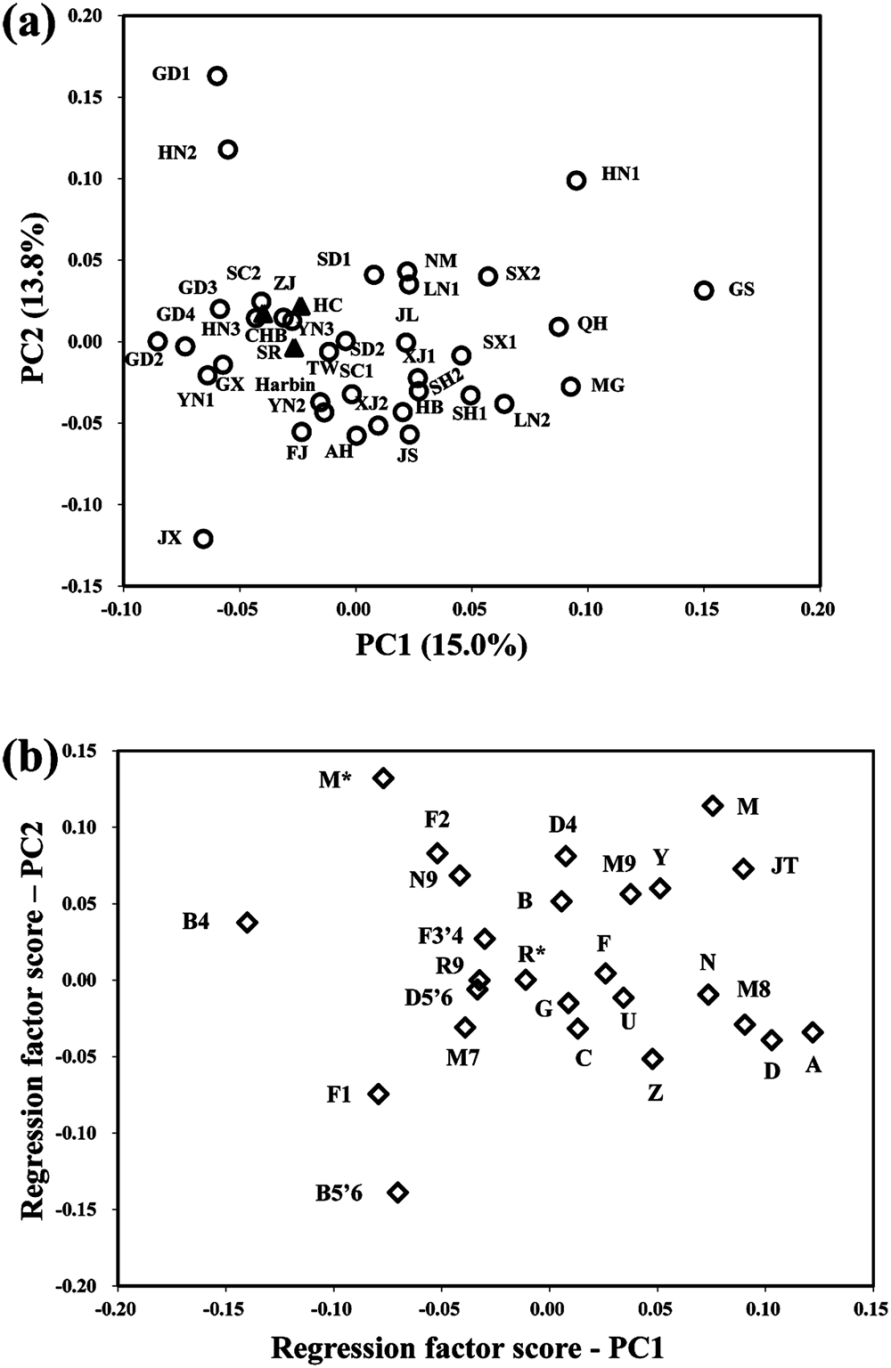
Principal component analysis of CHBs, SRs and HCs from Yunnan province in Southwest China and the previously reported Han Chinese populations across China. (**a**) PC map of Han regional populations based on mtDNA haplogroup frequencies. Sample sets in this study were marked by solid triangles. The reported Han Chinese populations (data shown in Supplementary Table S3) were marked by hollow circles. The abbreviations of Han regional populations were the same with those in Supplementary Table S3. (**b**) Plot of mtDNA haplogroup contribution to the first and second PCs. Abbreviations: PC, principal component.

According to the obtained mtDNA variants information, all 860 individuals could be classified into definite haplogroups, with only a few lineages showing unassigned M and R status (Supplementary Table S1). Principal component results obtained from the mtDNA haplogroup frequency distribution showed that the CHB, SR and HC samples from Yunnan Province were closely clustered (Fig. 1), indicating no apparent population stratification among our study samples.

Among these prevalent haplogroups (each was shared by at least 3% individuals in each population) listed in Table 2, haplogroup D5 had a significantly higher frequency distribution in CHBs than in HCs (*P* = 0.017, OR = 2.87, 95% confidence interval [CI] = 1.21–6.81) and SRs (*P* = 0.049, OR = 2.90, 95% CI = 1.01–8.35). The other haplogroup frequencies showed no significant difference between CHBs and HCs/SRs. There was also no significant difference in the haplogroup frequencies between SRs and HCs (Table 2). With HCs and SRs pooled together, the association of D5 and chronic HBV infection was highly significant (*P* = 0.008, OR = 2.81, 95% CI = 1.32–6.00) (Table 3). Since HBeAg is an indicator of active replication of HBV in infected patients, and serological conversion of HBeAg positive to HBeAg negative is of great clinical significance, we divided the overall CHB patients into two groups, 62 HBeAg positive (HBeAg (+)) and 210 HBeAg negative (HBeAg (−)), to investigate whether mtDNA haplogroup is associated with HBeAg sero-status among CHBs. No association was observed between mtDNA haplogroup and HBeAg status (Table 4), possibly because of the limited number of samples in this analysis. Overall, these results indicated an association between mtDNA haplogroup D5 and chronic HBV infection.

**Table 2:** Haplogroup frequencies in 272 individuals with chronic HBV infection, 278 spontaneously recovered subjects and 310 healthy controls.

Haplogroup^a^			CHB vs. HC				CHB vs. SR				SR vs. HC			
			CHB	HC	*P* value^b^	OR (95% CI)	CHB	SR	*P* value^b^	OR (95% CI)	SR	HC	*P* value^b^	OR (95% CI)
M*			22	27	0.95	0.98 (0.48–2.00)	22	23	0.86	0.93 (0.42–2.08)	23	27	0.68	1.18 (0.55–2.52)
M7			18	26	0.81	0.91 (0.44–1.91)	18	26	0.18	0.57 (0.25–1.29)	26	26	0.28	1.52 (0.71–3.26)
M8			24	35	0.72	0.88 (0.45–1.74)	24	28	0.43	0.74 (0.34–1.59)	28	35	0.96	1.02 (0.50–2.10)
D			46	38	0.15	1.57 (0.85–2.89)	46	42	0.87	1.06 (0.54–2.08)	42	38	0.12	1.72 (0.87–3.41)
	D4		19	26	0.74	0.88 (0.41–1.87)	19	19	0.86	0.92 (0.39–2.19)	19	26	0.82	1.10 (0.49–2.49)
	D5		22	10	0.017	2.87 (1.21–6.81)	22	9	0.049	2.90 (1.01–8.35)	9	10	0.59	1.36 (0.44–4.19)
A			19	32	0.36	0.71 (0.35–1.47)	19	21	0.63	0.82 (0.35–1.88)	21	32	0.77	1.12 (0.52–2.40)
R9			53	51	0.55	1.19 (0.67–2.14)	53	64	0.54	0.82 (0.43–1.56)	64	51	0.10	1.68 (0.90–3.13)
	F		46	47	0.76	1.10 (0.60–2.01)	46	51	0.71	0.88 (0.45–1.72)	51	47	0.31	1.40 (0.74–2.66)
		F1	30	27	0.60	1.20 (0.60–2.41)	30	38	0.46	0.76 (0.36–1.59)	38	27	0.14	1.72 (0.84–3.53)
B			46	53	0.78	1.09 (0.61–1.96)	46	42	0.91	0.96 (0.49–1.90)	42	53	0.59	1.20 (0.63–2.29)
	B4		24	29	0.90	1.04 (0.52–2.10)	24	23	0.65	0.83 (0.38–1.84)	23	29	0.42	1.36 (0.64–2.90)
	B5		17	19	0.71	1.16 (0.53–2.54)	17	16	0.71	1.19 (0.48–2.92)	16	19	0.99	0.99 (0.41–2.39)
others			44	48	—	—	44	32	—	—	32	48	—	—

^a^Haplogroups with large sample size are presented in a nest clade. Haplogroup D contains D4 and D5; F1 belongs to F, and F belongs to R9; and B contains B4 and B5.

CHB, chronic HBV infected; HC, healthy control; SR, spontaneously recovered.

OR, odds ratio; CI, confidence interval.

^b^*P* values, ORs and 95% CIs between each two groups were calculated by binary logistic regression adjusting for gender and age.

**Table 3:** Haplogroup frequencies in 272 CHBs and 588 combined controls (278 SRs and 310 HCs).

Haplogroup^a^			CHB	Control	*P* value^b^	OR (95% CI)
M*			22	50	0.89	0.96 (0.50–1.82)
M7			18	52	0.47	0.78 (0.40–1.53)
M8			24	63	0.65	0.87 (0.47–1.60)
D			46	80	0.31	1.32 (0.77–2.27)
	D4		19	45	0.64	0.85 (0.43–1.69)
	D5		22	19	0.008	2.81 (1.32–6.00)
A			19	53	0.37	0.74 (0.38–1.43)
R9			53	115	0.91	0.97 (0.58–1.64)
	F		46	98	0.90	0.97 (0.56–1.66)
		F1	30	65	0.84	0.94 (0.51–1.73)
B			46	95	0.93	1.03 (0.60–1.75)
	B4		24	52	0.89	0.96 (0.51–1.80)
	B5		17	35	0.72	1.14 (0.56–2.31)
others			44	80	—	—

^a^Haplogroups with large sample size are presented in a nest clade. Haplogroup D contains D4 and D5; F1 belongs to F, and F belongs to R9; and B contains B4 and B5.

CHB, chronic HBV infected; HC, healthy control; SR, spontaneously recovered.

OR, odds ratio; CI, confidence interval.

^b^*P* values, ORs and 95% CIs were calculated by binary logistic regression adjusting for gender and age.

**Table 4:** Haplogroup frequencies in 62 HBeAg positive and 210 HBeAg negative HBV infected individuals.

Haplogroup^a^			HBeAg (+)	HBeAg (−)	*P* value^b^	OR (95% CI)
M*			7	15	0.29	1.67 (0.66–4.23)
M7			2	16	0.39	0.42 (0.10–1.87)
M8			8	16	0.21	1.81 (0.75–4.36)
D			15	31	0.09	1.81 (0.93–3.51)
	D4		5	14	0.78	1.26 (0.44–3.58)
	D5		7	15	0.29	1.67 (0.66–4.23)
A			5	14	0.78	1.25 (0.44–3.58)
R9			9	44	0.38	0.68 (0.32–1.45)
	F		9	37	0.71	0.83 (0.39–1.79)
		F1	5	25	0.65	0.68 (0.25–1.82)
B			9	37	0.71	0.83 (0.39–1.79)
	B4		4	20	0.62	0.68 (0.23–2.05)
	B5		4	13	1.00	1.07 (0.34–3.37)
Others			7	37	—	—

^a^Haplogroups with large sample size are presented in a nest clade. Haplogroup D contains D4 and D5; F1 belongs to F, and F belongs to R9; and B contains B4 and B5.

OR, odds ratio; CI, confidence interval.

^b^Two tailed *P*-value of Fisher’s exact test.

**Figure 2:**
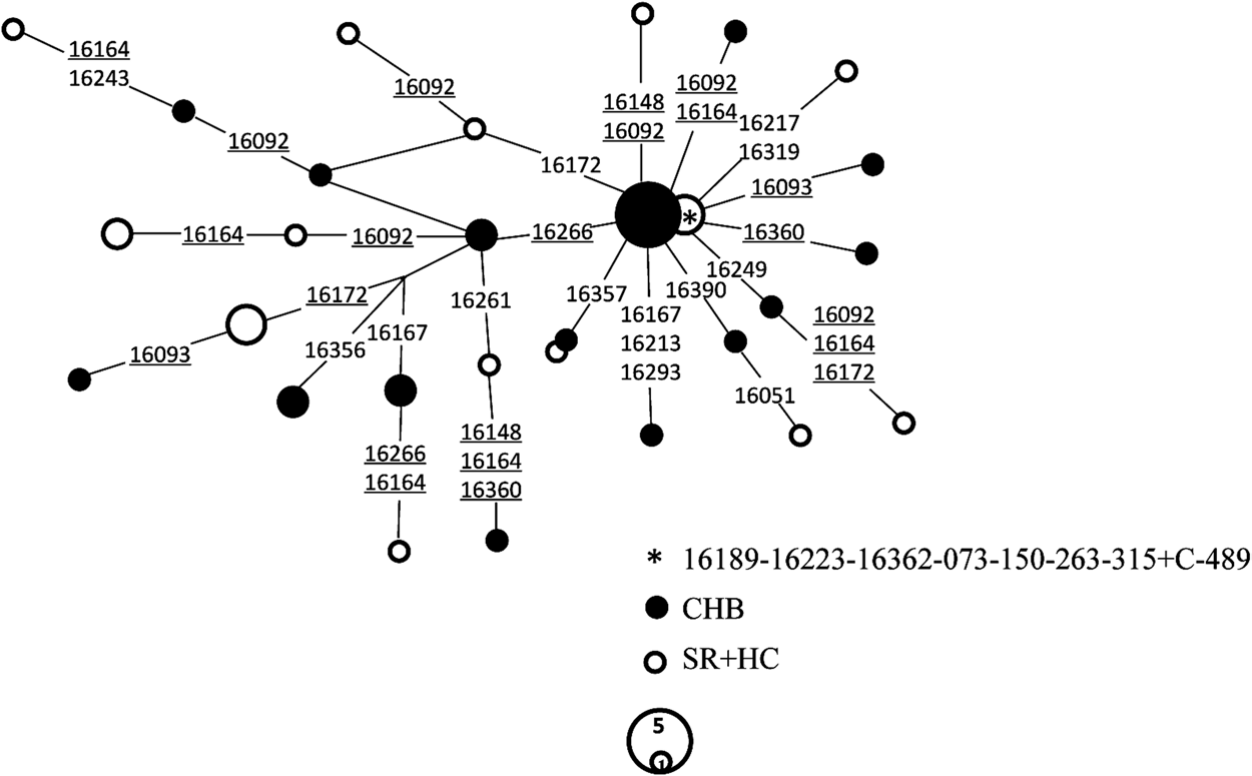
Network of haplogroup D5 in CHBs and combined controls consists of SRs and HCs. The variant order of the mtDNA control region is arbitrary on the branch. Each circle represents a kind of mtDNA haplogroup. The area of each circle is proportional to the haplogroup frequency. Sample sizes of the smallest and the largest sub-lineages were labeled in the corresponding circles. Length mutation of C-tract in region 16184–16193 was not considered during the network construction. The asterisks denote ancestral nodes of haplogroup D5.

The network of haplogroup D5 constructed based on variants of the hypervariable segment 1 (HVS-I) (np16024–16400) of the mtDNA control region revealed a distinct distribution pattern between CHBs and the combined controls comprising SRs and HCs. Only two of 27 (7.4%) mtDNA haplotypes belonging to D5 were shared between CHBs and non-CHB controls (Fig. 2).

### Characterisation of clinical parameters in CHBs according to haplogroups

**Figure 3:**
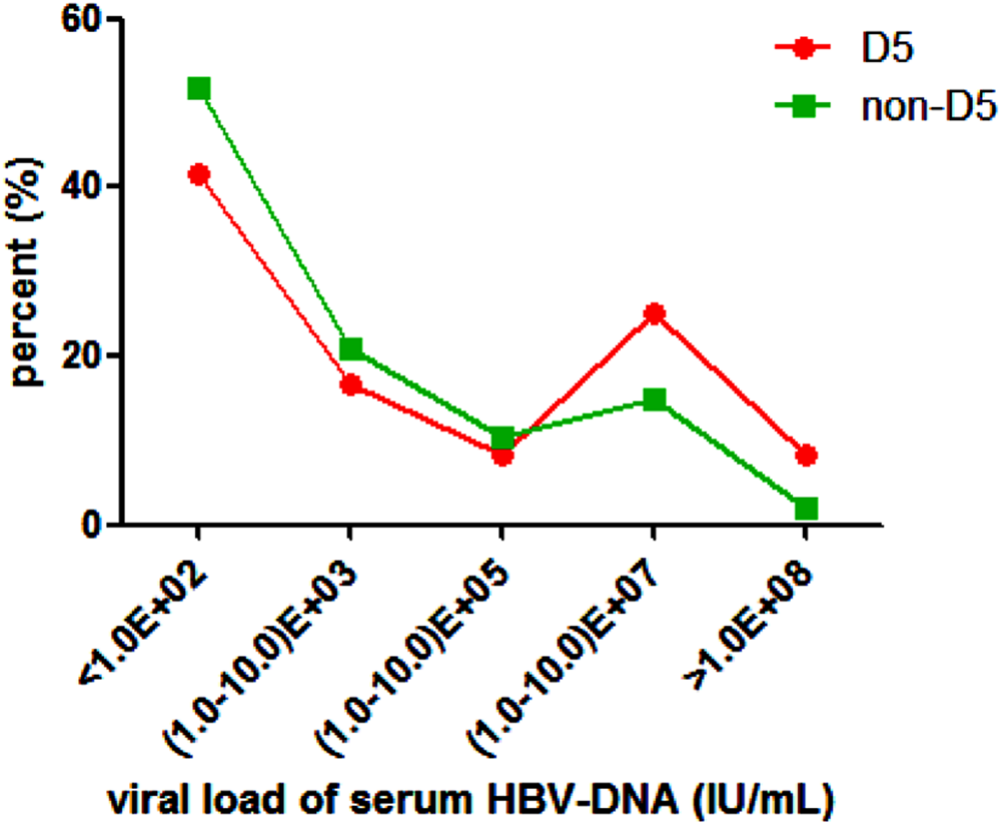
Viral load of serum HBV-DNA in CHBs belonging to haplogroup D5 and non-D5. We divided the viral load into five categories arbitrarily. The horizontal axis represents the viral load of serum HBV-DNA. The vertical axis represents the proportion of participants whose viral load falls in the respective viral load categories among CHBs with D5 or other haplogroup backgrounds.

**Figure 4:**
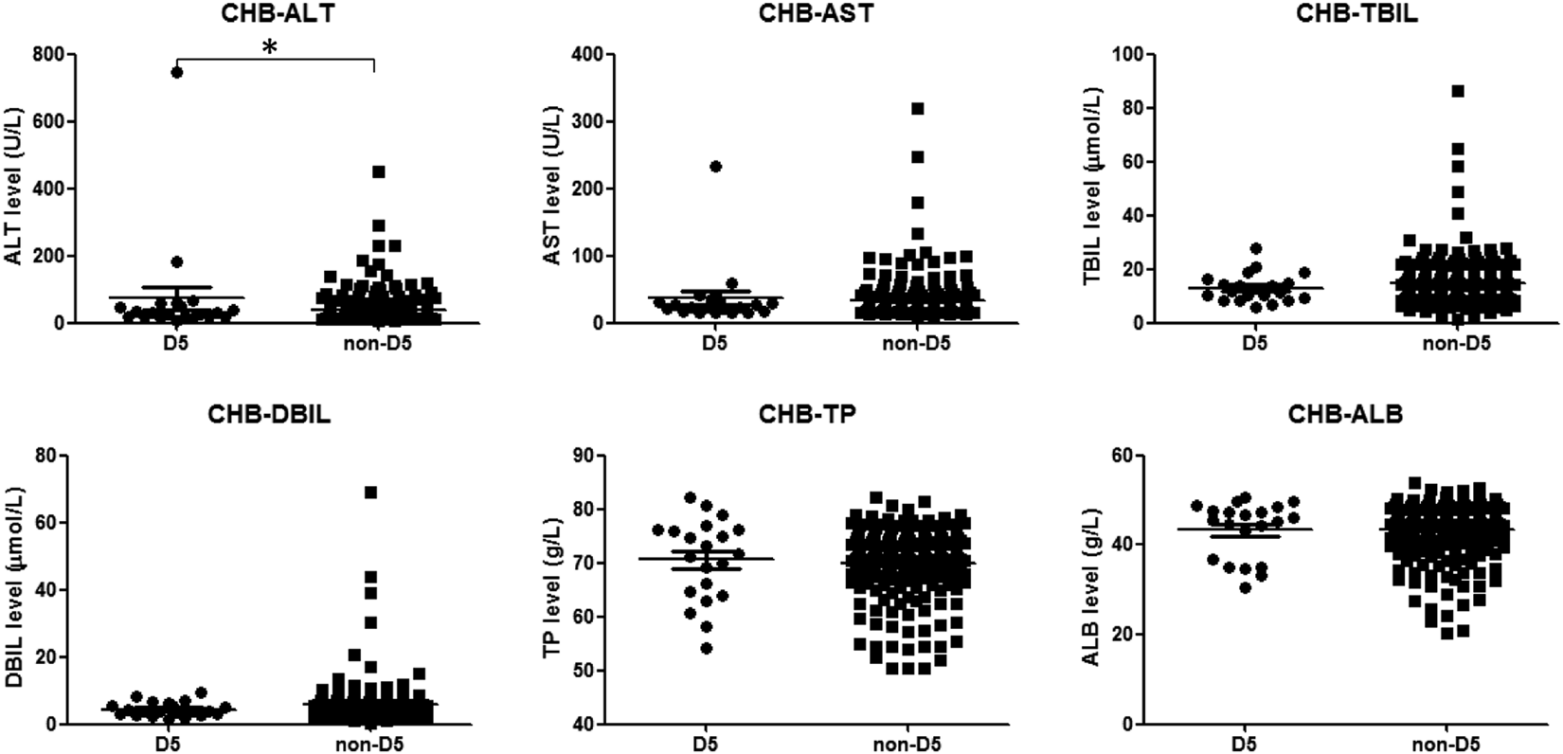
Clinical parameters of liver function in CHBs belonging to haplogroup D5 and non-D5. Serum levels of ALT (alanine transaminase), AST (aspartate aminotransferase), TBIL (total bilirubin), DBIL (direct bilirubin), TP (total protein) and ALB (albumin) were compared between D5 and non-D5 haplogroup members. Horizontal lines across the plots mark the mean expression level. Asterisk indicates significant difference (*P* < 0.05).

As haplogroup D5 showed a higher frequency in CHBs, we conducted further tests to see whether this haplogroup influences HBV viral load (VL) or indices of liver function. First, we screened the samples from the CHBs belonging to haplogroup D5 (n = 12) and non-D5 (n = 154) for VL based on HBV-DNA. We categorized the CHB individuals according to five VL degrees. The distribution of VL showed that the proportion of CHBs in haplogroup D5 was less than that in non-D5 when the VL was below 1.0 × 10^6^. In the contrary, the proportion of individuals in haplogroup D5 was higher than that in non-D5 in the topmost VL degree (VL > 1.0 × 10^7^) (Fig. 3). We then compared the serum ALT, AST, TBIL, DBIL, TP and ALB levels of CHBs belonging to haplogroup D5 (n = 21) and nonD5 (n = 249 for ALT, AST, TBIL and DBIL; n = 242 for TP and ALB). The overall difference in ALT levels between D5 and non-D5 CHBs was statistically significant (*P* = 0.02), with the highest ALT level observed in a patient belonging to haplogroup D5. However, when we excluded the highest ALT from D5, the overall difference was not statistically significant. The levels of the other five parameters were not different between D5 and non-D5 CHBs (Fig. 4).

## Discussion

Chronic hepatitis B, caused by HBV infection, is one of the most prevalent infectious diseases globally. The mechanisms underlying acute HBV infection or its progression to chronicity remain largely unknown. It was reported that the outcome of HBV infection does not appear to be determined by variations in the virulence of viral strains, and variation in host genes may partly explain the variability in HBV infection outcomes or the molecular mechanisms of viral clearance^27,28^. Instead, host factors are more likely to affect the disease outcome^11,29,30^. Recently, many researchers have tried to investigate host genetic factor that may explain the molecular pathogenesis of HBV infection. These include several genome wide association studies (GWASs) that have identified single nucleotide polymorphisms (SNPs) at several loci linking genetic susceptibility to HBV infection among populations living in HBV endemic areas. Following the first GWAS that investigated host genetic factors associated with chronic hepatitis B in Japan^31^, other GWASs have been carrying out in China and Korean in succession. Some risk factors such as rs3077 and rs9277535 in *HLA-DP*, as well as rs2856718 and rs7453920 in *HLA-DQ* at 6p21.32 were highly replicated in GWASs^9,11,12,32,33^. At the same time, some new loci were also identified, for instance, the *INST10* gene at 8p21.3 and its eQTL SNP rs7000921^9^, indicating that there may be other genetic factors yet to be discovered which contribute to HBV infection and its overall pathology.

In recent years, mitochondrial function, mtDNA variants and mtDNA haplogroup have been widely investigated in many kinds of human diseases^18,34–37^ including viral infectious diseases^20,38,39^. Relationship between low circulating mtDNA contents and increased risk of cirrhosis in HBV infected individuals has been reported^40^. The rate of D-loop mutations was significantly higher in hepatocellular carcinoma (HCC) individuals with HBV infection than in normal individuals^41^. Additionally, several studies have discovered interactions between HBV protein HBx and mitochondria which affects mitochondrial functionality^22,24,25^.

Although chronic HBV infections are widely spread across the globe, the burden of the disease is disproportional, with places like China bearing the heaviest brunt. We hypothesized that the geographic distribution of mtDNA haplogroup may be associated with this endemicity. In this study, we analysed mtDNA haplogroup distribution in 272 CHB patients, 278 SRs and 310 HCs from Yunnan Province in Southwest China. Our results indicated that mtDNA haplogroup D5 may confer genetic susceptibility to chronic HBV infection (Table 2). When SRs and HCs were put together as a combined control, the risk effect of haplogroup D5 for chronic HBV infection was consistently robust (Table 3). Principal component analysis (PCA) showed that the three sample groups were clustered together, suggesting a limited interference from any sampling bias or population stratification on behalf of mtDNA inheritance.

A large amount of association studies suggested an association between mtDNA haplogroup and many human diseases such as Leber hereditary optic neuropathy (LHON)^42,43^, sepsis^44^ and type 2 diabetes mellitus^45,46^. These studies further showed that the associations were always accompanied by geographically-based diversities. MtDNA haplogroups were also investigated in outcomes of human immunodeficiency virus (HIV) infection and antiretroviral therapy. Haplogroup H, I and K were associated with increased lipoatrophy in European-American and European individuals taking antiretroviral medication. Haplogroup H was also associated with lower likelihood of AIDS progression in Spanish individuals. Several anti-HIV drugs have been reported to induce mitochondrial toxicity^47^. Our results suggested that haplogroup D5 could be a risk factor for chronic HBV infection in Yunnan Province, Southwest China. However, enrichment of longevity phenotype in haplogroups D4b2b, D4a and D5 in the Japanese population was also reported^48^. Haplogroup D5 was also associated with esophageal cancer in Han Chinese from the Taihang Mountain area and Chaoshan area^49^. Transmitochondrial cytoplasmic hybrid (cybrid) cells harboring haplogroup D5 and F were associated with an increased risk of type 2 diabetes^45^. These reports revealed the role of haplogroup D5 as well as other mtDNA haplogroups more complicate in human diseases.

Interactions between HBeAg and the host were complex during HBV infection. HBeAg may impair both innate and adaptive immune response to promote chronic HBV infection, but the role of HBeAg in natural infection remains largely unknown^50,51^. Although our results showed haplogroup D5 as a risk factor for chronic HBV infection, we did not observe association between mtDNA haplogroup and HBeAg status in patients chronically infected with HBV, indicating that mtDNA haplogroup may not contribute to disease progression after chronic HBV infection. Mixed results have also been seen in mtDNA haplogroups and diseases. For instance, European mtDNA haplogroups were associated with insulin resistance and atherogenic dyslipidemia^52^ but was not with hepatitis C virus (HCV) treatment response in HIV/HCV-coinfected patients^53^. A common European haplogroup, J, has been reported to be protective against Parkinson’s disease^54^ but increase the phenotypic expression of certain mutations of LHON^55^. These reports demonstrated that the role of mtDNA haplogroup in disease risk may differ in various populations, possibly influenced by environment, ethnicity, gender, age of infection onset, nuclear gene mutation, as well as coefficient of genetic variants.

We also found that CHBs belonging to haplogroup D5 showed a trend towards having a very high VL and a lower frequency of VL <100 IU/mL (Fig. 3), which was considered as the cut-off for clinical HBV-DNA negativity. All patients with a HBV DNA >20000 IU/ml are offered treatment clinically due to the strong correlation between high viremia, cirrhosis, and hepatocellular carcinoma (HCC)^56^. Among our patients who underwent serum quantification of HBV DNA, there were 5 of 12 (41.67%) in D5 CHBs and 41 of 154 (26.62%) in non-D5 CHBs with a HBV DNA >20000 IU/ml. This indicates a high occurrence of active HBV DNA replication among D5 CHBs. ALT is the most commonly used liver function parameter. Acute liver injury can be diagnosed by the increased level of ALT above 10 times of the upper limit of normal range (ULN, reference value as 0–40 U/L according to our hospital-based test)^57,58^. Only one out of 22 (4.55%) D5 CHBs and one out of 249 (0.40%) non-D5 CHBs who underwent ALT quantification had ALT >10 ULN (748 U/L and 451 U/L respectively). After excluding the highest ALT in D5 CHBs, the overall ALT did not significantly vary between haplogroup D5 and non-haplogroup D5 CHBs. This outcome was likely affected by the limited number of D5 participants available for analysis. Other liver function parameters including AST, TBIL, DBIL, TP and ALB showed no difference between D5 and non-D5 CHBs. These results further reinforced the likely role of haplogroup D5 as a risk for chronic HBV infection, but should be received with caution. Since D5 CHBs is a small group, the distribution of clinical parameters such as HBV DNA VL and ALT level were not well-proportioned, hence the high VL and ALT we observed in D5-CHBs may be occasional. Further validation using more clinical data of D5 is needed.

Antiviral drugs such as lamivudine and telbivudine may decrease mtDNA content measured by mtDNA copy number and affect mitochondrial function^59^. The insufficient information of drug treatment limited us from evaluating the mtDNA difference among HBV infected subpopulations and other aspects of mtDNA. Therefore, future study designs should also consider additional clinical aspects like antiviral drug treatment, HBV infection period and transmission routes. The limited sample size in this study, especially after dividing the overall CHBs into smaller categories, could affect the evaluation of clinical parameters. More samples are needed to investigate whether haplogroup D5 is associated with clinical parameters of liver function in CHBs. Lastly, the unavailable cell line of haplogroup D5 barred us from further detecting the influence of different matrilineal background on HBV infection. Functional assays to verify mtDNA haplogroup or certain mtDNA variation(s) in chronic HBV infection based on applicable cell line is needed.

In summary, we detected mtDNA haplogroup distribution in a general population which is composed of CHBs, SRs and HCs from Yunnan Province in Southwest China. We observed higher frequency distribution of haplogroup D5 in chronic HBV infected individuals than in SRs and HCs. The network results revealed a distinct distribution pattern of haplogroup D5 between CHBs and combined controls. Moreover, whether haplogroup D5 may confer risk to liver injury after chronic HBV infection needs to be solidified by more D5 samples. Future studies with larger sample size and functional assessment will be essential to identify the role of haplogroup D5 in chronic HBV infection.

## Materials and Methods

### Subjects

We enrolled 272 chronic HBV infected individuals (CHB), 278 spontaneously recovered (SR) subjects and 310 healthy controls (HC) who had never been infected by HBV. All of the HBV infected individuals were diagnosed with hepatitis B surface antigen (HBsAg) positive for at least 6 months. Patients with HCV or HDV infection and alcoholic liver, fatty liver or autoimmune liver disease were excluded from enrollment. Those who were negative for HBsAg and positive for both anti-HBs and anti-HBc were defined as SRs. The SR and HC subjects were adults who come to hospital for physical examination. Recruitment criteria for each group were listed in Supplementary Table S2. Peripheral blood and clinical information including serum levels of HBV-DNA VL, ALT, AST, TBIL, DBIL, TP and ALB were collected. All of the individuals are from Yunnan province in Southwest China. Written informed consents conforming to the tenets of the Declaration of Helsinki were obtained from all participants prior to enrollment into this study. The experimental methods were carried out in accordance with the approved guidelines and regulations. All experimental protocols were approved by the institutional review board of the Second People’s Hospital of Yunnan Province.

### mtDNA control region sequencing and haplogroup classification

Genomic DNA was extracted from the peripheral blood using EX-DNA whole blood genome kit (Suzhou Tianlong Bio-technology Co., Ltd.) by automatic Nucleic Acids Extraction System NP968 (Xi’an Tianlong Science & technology Co., Ltd.) according to the manufacturer’s instruction. The mtDNA control region sequence was amplified by using primer pair (L15575, 5′-ACACAATTCTCCGATCCGTC-3′ and H575, 5′-TGAGGAGGTAAGCTACATAAACTG-3′). PCR reactions were performed in a 50 μL reaction volume containing 45 μL of Jinpai MIX (green) (Beijing TsingKe Biotech co., Ltd.), 10 μM of each primer, and ~50 ng genomic DNA. The PCR condition was run under the following procedures: a pre-denaturation cycle of 98 °C for 2 min; 30 amplification cycles of 98 °C for 10 s, 57 °C for 15 s, and 72 °C for 30 s; and a final extension cycle of 72 °C for 5 min. PCR products were purified and analysed by the ABI PRISM TM 3730 × DNA analyzer (Applied Biosystems). Sequencing primers we used were as follows: L15996, 5′-CTCCACCATTAGCACCCAAAGC-3′; L29, 5′-GGTCTATCACCCTATTAACCAC-3′; L16209, 5′-CCCCATGCTTACAAGCAAGT-3′; H16347, 5′-GGGGACGAGAAGGGATTTGA-3′ and H575. For samples which could not be accurately classified based on the mtDNA control region sequence variation, a fraction of mtDNA-coding region was sequenced to justify the classification. Most of the primers used in this study were reported previously^34,60^.

Sequence variants of each sample were recorded based on the revised Cambridge Reference Sequence (rCRS)^61^. Each mtDNA haplogroup was assigned according to the latest version of the phylogenetic tree of human mtDNA (mtDNA tree Build 17, http://phylotree.org/tree/index.htm)^62^ and reconfirmed by the web-based bioinformatics platform mitotool (http://www.mitotool.org/)^63^.

### Statistical analyses

Clinical information including gender, age, serum levels of ALT, AST, TBIL, DBIL, TP and ALB were compared in SPSS 17.0 and the PRISM software (GraphPad Software, Inc, La Jolla, CA, USA). We also compared the VL of HBV-DNA and ALT, AST, TBIL, DBIL, TP and ALB levels between CHBs with the haplogroup associated with chronic HBV infection and other haplogroups. Chi-square tests and unpaired student’s t test were used to examine the differences in clinical characters of participants. A *P* value < 0.05 was considered statistically significant. To examine the similarity of mtDNA genetic structures of our CHB, SR and HC sample sets, PCA was performed based on the mtDNA haplogroup distribution frequencies using the POPSTR software (Henry Harpending, 1997) incorporating published Han Chinese data from different regions across China. The frequencies of 26 main haplogroups collected from 36 published data sets together with our samples were used to conduct PCA. The published mtDNA data sets reanalysed in this study were shown in Supplementary Table S3.

To avoid potential bias caused by small sample size and to ensure the statistical power, only haplogroup frequencies shared by more than 3% of individuals in each subset of our cohort were considered to conduct statistical analysis. Due to the unmatched gender and age of SRs to the other two groups, potential association between mtDNA haplogroups and HBV infection was estimated by binary logistic regression with an adjustment for gender and age in each two groups. We further compared the mtDNA haplogroup frequencies in HBeAg positive and HBeAg negative patients by using the Fisher’s exact test (two tailed), considering that there were cases with cell counts below five.

Median-joining network for these mtDNAs belonging to the chronic HBV infection associated haplogroup based on the association analysis was constructed by Network 5.0.0.1 (http://www.fluxus-engineering.com/sharenet.htm)^64^. Considering that a wider range of variants may increase the complexity of the topological structure of the network, we used just the variants distributed in the HVS-I of mtDNA control region during the network construction. Due to the relatively small sample size of haplogroup D5, we put SRs and HCs together as a non-CHB control group to draw the network.

## Electronic supplementary material

Supplementary information
