# Supplementary material for: Correlations between mitochondrial DNA haplogroup D5 and chronic hepatitis B virus infection in Yunnan, China
Source: Sci Rep. 2018 Jan 17;8:869. doi: 10.1038/s41598-018-19184-6 (PMC5772044; doi:10.1038/s41598-018-19184-6)
Supplement: Supplementary file 1 — Supplementary information [file 41598_2018_19184_MOESM1_ESM.pdf]

Supplementary Information

Correlations between mitochondrial DNA haplogroup D5 and chronic hepatitis B virus infection in Yunnan, China

Xiao Li [1], Tai-Cheng Zhou [1], Chang-Hui Wu [1], Li-Lin Tao [1], Rui Bi [2], Li-Jun Chen [1], De-Yao Deng [3], Chang Liu [3], Newton O. Otecko [4,5], Yang Tang [6], Xin Lai [1], Liang Zhang [1,*] and Jia Wei [1,*]

[1] Central Lab, Liver Disease Research Center, the Second People's Hospital of Yunnan Province, Kunming 650203, Yunnan, China.

[2] Key Laboratory of Animal Models and Human Disease Mechanisms of the Chinese Academy of Sciences and Yunnan Province, Kunming Institute of Zoology, Kunming 650223, Yunnan, China.

[3] Clinical Laboratory of the Second People's Hospital of Yunnan Province, Kunming 650203, Yunnan, China.

[4] State Key Laboratory of Genetic Resources and Evolution & Yunnan Laboratory of Molecular Biology of Domestic Animals, Kunming Institute of Zoology, Chinese Academy of Sciences, Kunming 650223, China.

[5] Kunming College of Life Science, University of Chinese Academy of Sciences, Kunming 650204, China.

[6] The first affiliated hospital of kunming medical university, Kunming, Yunnan Province, 650000,China.

Xiao Li and Tai-Cheng Zhou contributed equally to this work.

Corresponding authors at: Liver Disease Research Center, the Second People's Hospital of Yunnan Province, Kunming 650203, Yunnan, China. Tel.: +86 087165156658; fax: +86 087165156658. E-mail addresses: weijia19631225@163.com (Jia Wei), or liangz_tkl@163.com (Liang Zhang).

Supplementary Table S1. mtDNA sequence variations of 272 CHB patients, 278 SRs and 310 HCs in this study.

Sample	Haplogroup	Region 1 (16000+) [a]	Region 2 [a]	4866-5461 [b]
chronic HBV infected samples				
HB-52	A	192 223 290 319 362	73 235 263 315+C 523-524d	
HB-145	A	223 290 319 362	73 152 195 235 263 315+C	
HB-160	A	223 290 319 362	73 152 235 309+C 523-524d	
HB-165	A	129 223 290 319 362	73 152 235 263 309+2C 315+C 523-524d	
HB-208	A	086 150 223 290 319 362	73 152 235 263 309+C 315+C 523-524d	
HB-283	A	223 290 293C 319 519	73 235 263 309+C 315+C 523-524d	
HB-314	A	223 235 290 311 319 362 519	73 152 234 235 263 309+2C 315+C 523-524d	
HB-251	A11b	223 234 290 293C 319 519 527	73 152 235 263 309+C 315+C 523-524d	
HB-26	A13	124 223 290 319 362	73 152 200 235 263 309+C 315+C 523-524d	
HB-245	A13	124 223 290 319 362	73 152 200 235 263 309+2C 315+C 523-524d	
HB-48	A15	223 290 319 362	73 152 204 207 235 309+2C 315+C 523-524d	
HB-65	A15	223 261 290 319 362	73 152 200 207 235 309+2C 315+C 523-524d	
HB-140	A15	207 223 290 319 362	73 152 207 235 309+C 315+C 523-524d	
HB-201	A15	223 290 319 362	73 152 207 235 315+C 523-524d	
HB-206	A15	223 290 319 362	73 152 204 207 235 309+2C 315+C 523-524d	
HB104	A15b	093 172 223 234 290 319 362	73 152 207 235 309+C 315+C	
HB-311	A19	223 235 290 311 319 362 519	73 152 199 234 235 263 309+C 315+C 523-524d	
HB-144	A25	179 223 290 311 319 362	73 152 235 263 315+C 522 523-524d	
HB-53	A5b	093 126 223 234 235 290 319 519	73 152 235 263 309+C 315+C 523-524d	
HB133	B	182C 183C 189 234 243 463 519	73 94 103 131 146 263 309+C 315+C 481 523-524d	
HB139	B	183C 189 189+C 217 234 319 519	73 263 309+C 315+C	
HB-2	B4	189 189+C 217 274 519	73 146 150 152 263 309+C 315+C 573+C	

HB-11	B4	183C 189 207 217d 218+C 261 354	73 263 309+2C 315+C 523-524d	
HB-69	B4	182C 183C 189 217 261 357 519	73 263 309+2C 315+C 523-524d	
HB-70	B4	093 182C 183C 189 217 240 261	73 195 263 309+C 315+C 523-524d	
HB-92	B4	189 217 311 362 519	73 146 263 315+C 523-524d	
HB107	B4	182C 217 261 362 399 519	73 189 263 309+2C 315+C 523-524d	
HB134	B4	183 189 217 362 519	73 152 263 309+2C 316C 316+A	
HB-146	B4	183C 189 217 519	73 152 207 263 309+C 316C 316+A	
HB-176	B4	129 182C 183C 189 217 261	73 146 263 309+2C 315+C 523-524d	
HB-203	B4	182C 183C 189 217 519	73 200 263 316C 316+A 524+AC	
HB-204	B4	182C 183C 189 217 274 304 335 519	73 146 150 263 309+2C 315+C	
HB-289	B4	093 182C 183C 189 217 240 261	73 195 263 309+C 315+C 523-524d	
HB-304	B4	126 182C 183C 189 217 261 519	73 207 263 315+C 523-524d	
HB-41	B4a	189 217 261 266 519	73 146 263 316+C	
HB-150	B4a	182C 183C 189 217 261 327 519	73 146 263 309+2C 315+C	
HB-152	B4a	182C 183C 189 217 311 362 519	73 146 263 315+C 523-524d	
HB-42	B4b	086 136 183C 189 217 301 519	73 146 207 263 309+C 315+C 513+CA	
HB-205	B4c1b2a	140 182C 183C 189 217 274 335 519	73 146 150 263 309+2C 315+C	
HB-F010	B4c1b2a	140 182C 183C 189 217 274 311 335 519	73 146 150 263 315+C	
HB-240	B4c1b2a	140 182C 183C 189 217 274 335 519	73 146 150 263 309+2C 315+C	
HB-235	B4c2	004 147 183C 184A 189 217 235 519	73 263 309+2C 315+C 523-524d	
HB-231	B4g	181C 182C 183C 189 213 217 261 292 519	73 263 309 310 523-524d	
HB-277	B4g	181C 182C 183C 189 213 217 261 292 519	73 146 263 309+2C 315+C 523-524d	
HB-85	B4m	176 182C 183C 189 217 240 261 519	73 263 309+2C 315+C 523-524d	
HB-25	B5	189 234 266A 519	73 210 263 315+C 523-524d 709 750	
HB-50	B5	188 189 235 266A 327 519	73 210 263 309+C 315+C 523-524d	

HB128	B5	140 189 266 291 519	73 207 210 263 315+C 523-524d	
HB-213	B5	111 140 183C 189 234 242 243 463 519	73 103 131 263 309+C 315+C 523-524d 534A	
HB-270	B5	140 183C 189 243 519	73 103 143 195 263 315+C 523-524d	
HB-273	B5	093 140 183C 189 243 355 519	73 103 263 315+C 523-524d	
HB-276	B5	140 183C 189 243 355 519	73 103 263 309+2C 315+C 523-524d	
HB-84	B5a	140 183C 189 189+C 266A 519 527	73 150 210 263 309+2C 315+C 499 523-524d	
HB113	B5a	140 182C 183C 189 266A 519	73 210 263 315+C 523-524d	
HB-190	B5a	140 182C 183C 189 266A 519	73 210 263 309+2C 315+C 523-524d	
HB-195	B5a	140 183C 189 266A 519	73 210 263 315+C 523-524d	
HB-216	B5a	140 183C 189 266A 482 519	73 210 263 309+C 315+C 523-524d	
HB-272	B5a	140 183C 189 266A	73 210 263 309+C 315+C 523-524d	
HB-313	B5a	140 183C 189 266A 519	73 210 263 309+2C 315+C 523-524d	
HB-142	B5a1c1a1	140 183C 189 189+C 262 266A 519	64 73 210 263 309+2C 315+C 523-524d	
HB-147	B5a2a1a	140 187 189 256 266 519	73 93 210 263 315+C 523-524d	
HB-298	B5b	136 140 183C 189 243 311 519	73 146 195 204 263 315+C 523-524d	
HB-F002	B6a	093 179 182C 183C 189 342	73 150 263 315+C	
HB-263	B6a1	051 182C 183C 189 311 519	73 263 309+C 315+C 356+C	
HB-269	B6a1	051 086 183C 189 220 298 519	73 263 309+2C 315+C 356+C	
HB-4	C	223 298 327 519	73 146 249d 263 309+2C 315+C 489 750	
HB-14	C	223 298 327 519	73 195 200 204 249d 263 309+C 315+C 489	
HB-38	C	183C 189 223 298 327 519	73 249d 263 309+2C 315+C 489	
HB-217	C	051 183C 189 223 278 298 327 519	73 249d 263 315+C 489	
HB-221	C	223 298 327 519	73 249d 263 309+C 315+C 460 489	
HB-239	C	223 298 327 519	73 249d 263 309+C 315+C 460 489	
HB-247	C	223 298 327 519	73 249d 263 309+C 315+C 460 489	

HB-297	C	114A 223 298 327 519	73 249d 263 309+C 315+C 460 489	
HB-154	C4	223 298 327 519	73 249d 263 309+C 315+C 489	
HB-166	C4	223 270 298 327 519	73 146 249d 263 310 489	
HB-194	C4	223 298 327 519	73 249d 263 309+2C 315+C 489	
HB-228	C4a2c	223 298 327 354 357 519	47 73 249d 263 309+2C 315+C 489 523-524d	
HB-249	C4b8a	093 223 298 327 519	73 249d 263 310 466 489	
HB97	D	223 362	73 263 309+C 315+C 489	5178A
HB119	D	223 293T 362	73 263 309+C 315+C 489	5178A
HB-180	D	184 223 311 362 400 468 519	73 263 309+C 315+C 489	5178A
HB-233	D	223 316 362	73 183 263 315+C 489	5178A
HB-301	D	223 294+A 362	73 263 309+C 315+C 489	5178A
HB-18	D4	167 172 174 223 362	73 263 315+C 489 523-524d 750	5048 5178A 5262
HB-207	D4	184 223 311 362	73 152 263 309+C 315+C 489	5178A
HB-F009	D4	223 249 362	73 263 309+C 315+C 489	5178A 5231
HB-15	D4a	129 223 249 311 362	73 152 263 315+C 489 750	
HB-181	D4a	129 209 223 362 519	73 152 263 309+C 315+C 489	
HB-182	D4b2b	193 223 362 519	73 194 263 315+C 489 523-524d	
HB-12	D4e1a	092 223 295 362 519	73 94 263 309+C 315+C 489 750	
HB-91	D4e1a	092 223 362	73 94 263 309+C 315+C 489	
HB-179	D4g2	223 362 526	73 263 298 315+C 489 523-524d	
HB-40	D4g2a	223 274 311 362	73 263 298 315+C 489 750	
HB-292	D4g2a	223 274 362	73 263 298 309+C 315+C 489	
HB-54	D4j1b2	172 223 362	73 185 263 309+C 315+C 489	
HB-209	D4j3	184 223 311 362	73 152 263 315+C 489	
HB-241	D4j3	184 223 311 362	73 263 315+C 489	

HB116	D4j6	223 362 519	73 146 263 309+C 315+C 489	
HB-163	D4j9	093 223 286 362	73 263 315+C 489	
HB-175	D4q	223 256 311 362 519	73 200 263 315+C 489	
HB-199	D4q	223 256 311 362 519	73 200 263 315 489	
HB-282	D4q	223 256 311 362 519	73 200 263 315+C 489	
HB-20	D5	183C 189 223 266 362	73 146 150 263 315+C 489 523-524d 567+C 750	
HB-62	D5	189 192+C 223 266 362	73 150 263 315+C 489 523-524d	
HB123	D5	164 182C 183d 186 189 223 266 356 362	73 150 263 315+C 489 523-524d	
HB-141	D5	164 182C 183d 186 189 223 266 356 362	73 150 263 315+C 489 523-524d	
HB-169	D5	182C 183C 189 223 362	73 150 263 309+C 315+C 489 523-524d	
HB-184	D5	182C 183C 189 223 249 362	73 150 263 315+C 489 523-524d	
HB-202	D5	164 167 182C 183C 189 223 266 362 519	73 150 263 315+C 489 523-524d	
HB-253	D5	164 182C 183C 189 223 261 266 362 519	73 150 263 309+2C 315+C 489 523-524d	
HB-286	D5	164 167 182C 183C 189 223 266 362 519	73 146 150 189 195 263 315+C 489 523-524d	
HB-303	D5	092A 164 182C 183C 189 223 362	73 150 152 263 315+C 489 523-524d	
HB120	D5a2a	093 164 172 182C 183C 189 223 266 362	73 146 150 263 315+C 489 523-524d 573+C	
HB-237	D5a2a	172 182C 183C 189 223 266 362	73 150 263 294 309+C 315+C 489 523-524d	
HB-259	D5a2a2	092 172 182C 183C 189 223 266 362	73 150 263 309+C 315+C 456 489 523-524d	
HB-191	D5a3	183C 189 223 360 362 519	73 150 263 309+2C 489	
HB-177	D5b1b2	183C 189 223 357 362 519	73 150 263 309+2C 315+C 456 489	
HB-49	D5b1d	189 223 362	73 146 150 152 263 309+C 315+C 489	
HB-83	D5b1d	182C 183C 189 223 362	73 146 150 263 309+2C 315+C 456 489	
HB-95	D5b1d	182+C 183C 189 223 362	73 146 150 263 309+C 315+C 456 489	
HB126	D5b1d	093 183C 189 223 362	73 146 150 263 309+2C 315+C 456 489	
HB-250	D5b1d	183C 189 223 362	73 146 150 263 315+C 456 489	

HB-260	D5b4	189 213 223 362 519	73 146 150 309+2C 315+C 456 489	
HB-155	D5c	188+C 189+C 293C 362 390 519	73 146 150 151 152 182 217 263 315+C 489	
HB-F008	F	304	73 249d 263 309+C 315+C	
HB-21	F1	189 189+C 304 390 519	73 195 249d 263 309+C 315+C 368 523-524d	
HB-24	F1	129 162 172 304 519	73 249d 263 315+C 523-524d	
HB102	F1	129 172 304 519	73 249d 263 315+C 394 523-524d	
HB103	F1	182C 183C 189 304 311 519	73 249d 263 309+C 315+C 523-524d	
HB131	F1	129 162 172 189 304 311 519	73 249d 263 309+2C 315+C 523-524d	
HB137	F1	108 129 162 172 214 304 519	73 249d 263 315+C 523-524d	
HB-143	F1	189 189+C 304 519	73 146 249d 263 309+2C 315+C	
HB-168	F1	129 162 172 189 304 335 355 519	73 249d 263 315+C 523-524d	
HB-178	F1	183C 189 232A 249 304 519	73 152 249d 263 315+C	
HB-183	F1	129 172 304 519	73 249d 263 315+C 523-524d	
HB-196	F1	162 172 304 519	73 249d 263 309+C 315+C 515 516-517d	
HB-F007	F1	183d 189+C 232A 249 304 311	73 249d 263 523-524d	
HB-23	F1a	172 240d 304 465 519	73 249d 263 309+2C 315+C 521-524d 750	
HB-232	F1a	129 172 304 519	73 249d 263 315+C 523-524d	
HB-302	F1a	129 172 295 304 519	52 53 54T 71d 73 249d 263 309+C 315+C 318 523-524d	
HB-300	F1a1	129 162 172 304 519	73 94 249d 263 309+2C 315+C 523-524d	
HB-218	F1a1a	108 129 162 172 304	73 150 195 249d 263 315+C 523-524d	
HB-10	F1a1c	036+A 038d 129 162 172 304 519	73 249d 263 315+C 523d 548 750	
HB-200	F1a1c	129 162 172 304 311 519	73 249d 263 315+C 523-524d 548	
HB-214	F1a1d	129 162 172 304 399 519	73 249d 263 309+C 315+C 523-524d	
HB-27	F1a3a	093 129 172 304 311	73 249d 263 309+C 315+C 523-524d	
HB-94	F1a3a	129 172 304 311 519	73 249d 263 315+C 523-524d	

HB-171	F1c1a	111 129 304 519	73 152 234 249d 263 315+C 523-524d	
HB-198	F1c1a	092 111 129 304 519	73 152 234 249d 263 309+C 315+C 523-524d	
HB-93	F1c1a1	111 129 266 304 519	73 152 249d 263 309+C 315+C 523-524d	
HB112	F1c1a1	111 129 266 304 519	73 152 249d 263 315+C 523-524d	
HB-265	F1c1a1	111 129 266 304 519	73 114 152 249d 263 310 523-524d	
HB-294	F1c1a1	111 129 266 304 519	73 152 249d 263 309+C 315+C 523-524d	
HB-246	F1d	189 284 304 519	73 146 249d 263 309+2C 315+C 523-524d	
HB-315	F1d	183C 189 278 304 357 519	73 146 151 207 249d 263 308+T 315+C 523-524d	
HB-57	F2a	203 215 256 274 291 304 519	73 249d 263 309+C 315+C	
HB-236	F2a	093 203 291 304 519	73 249d 263 309+C 315+C	
HB-71	F2a1	203 304 519	73 249d 263 315+C 750	
HB-299	F2b1	092A 291 304	73 249d 263 309+C 315+C 523-524d	
HB-307	F2b1	092A 093 291 304	73 249d 263 309+2C 315+C 523-524d	
HB-225	F2c2	067 271 304	73 146 249d 263 309+C 315+C	
HB99	F2h	304	73 195 249d 263 315+C	
HB110	F2h	304	73 195 249d 263 315+C	
HB111	F2h	304	73 195 249d 263 315+C 589A	
HB-226	F2i	221 239 304 362 519	73 152 195 249d 263 275 309+C 315+C	
HB-7	F3a	093 111 192 249 298 355 362 390	73 153 207 249d 263 309+C 315+C 750	
HB-153	F3a	111 192 249 298 355 362 390	73 207 249d 263 315+C	
HB-278	F3a1	093 260 292A 298 355 362	73 207 249d 263 315+C	
HB-193	F3b1	093 220C 265 298 362	73 150 152 249d 263 310+T	
HB130	F4a1b	126 140 207 304 362 399	73 146 249d 263 309+C 316+C 317A 456+T	
HB-34	G	129 163 223 311 362	73 143 263 315+C 489 709 750	5108
HB-212	G1a1	223 256 325 362 422 519	73 150 263 309+C 315+C 489	

HB-13	G2	172 223 272 278 290 319 362 519	73 152 263 309+C 315+C 489 750	
HB-F004	G2a	223 227 278 362	73 309+C 315+C 489	
HB-F006	G2a	183C 189 223 278 362	73 260 263 315+C 489	
HB-224	G2a1d	189 223 269 278 362	73 260 263 284 309+2C 315+C 489	
HB-187	G2a2a	223 227 278 362	73 152 207 263 309+C 315+C 489 523-524d	
HB-290	G2a4	172 223 272 278 290 319 362 519	73 152 263 309+C 315+C 489	
HB-223	G3	086 223 274 362 519	73 259 263 315+C 489	
HB-63	M	129 223 311	73 143 152 263 309+C 315+C 489 597+T	
HB-234	M	223 362	73 263 315+C 489	
HB-268	M	295 319	73 146 199 263 309+C 315+C 489 523-524d	
HB-8	M*	066 093 223 311	73 189 263 309+C 315+C 489	
HB-66	M*	223 271 311 519	73 263 315+C 489	
HB-74	M*	093 223 311 362	73 263 309+C 315+C 489	
HB-76	M*	129 223 234 260 292 362	73 146 263 315+C 489 750	
HB101	M*	223	73 263 309+C 315+C 489	
HB108	M*	188 223 256 266 290 311 519	73 204 207 263 315+C 489	
HB136	M*	223 381	73 263 309+C 315+C 489	
HB-256	M*	223 249 362	73 152 263 309+C 315+C 489	
HB-271	M*	223 278 362	73 152 260 263 309+C 315+C 489	
HB-279	M*	223 362	73 152 263 315+C 489	
HB-287	M*	223 269 271 311	73 150 263 309+2C 315+C 489	
HB-310	M10a1	129 193 214 223 294 311 357 497	73 146 263 315+C 489 523-524d 573+2C	
HB-32	M11	189 223	73 200 215 263 309+C 315+C 318 326 455+T 489	
HB-F013	M11	173 223	73 146 196 198 200 263 315+C 318 326 489	
HB-285	M12a1	223 234 290 304 362	73 125 127 128 263 315+C 318 489 513-514d	

HB-173	M13a1b	145 148 188 189 223 381	73 152 263 315+C 489	
HB-288	M13a1b	145 148 188 189 223 381	73 152 263 315+C 489	
HB127	M33c	111 223 362 519	73 194 263 309+C 315+C 489 523-524d	
HB-28	M5	129 223 278 362	73 263 315+C 489 709 750	
HB-9	M7	129 192 223 297 442	73 150 199 263 309+C 315+C 489 750	
HB-72	M7	093 129 179T 192 223 297	73 150 199 263 309+C 315+C 489	
HB-284	M71a1a	223 269 271 311 342	73 150 151 263 309+2C 315+C 489	
HB-F011	M74a	093 209 223 311 362 381	63 64 66 73 150 215 217 263 315+C 489	
HB-230	M74a	093 223 311 362 381	63 64 66 73 215 263 315+C 489 523-524d	
HB-81	M7b	129 183C 189 189+C 223 297	73 150 199 204 263 309+2C 315+C 456 489	
HB-185	M7b	129 192 223 297	73 150 199 263 309+C 315+C 489	
HB-189	M7b	129 192 223 297	73 150 199 263 315+C 489	
HB-255	M7b	129 192 223	73 150 182 199 263 315+C 459d 489	
HB-211	M7b1a	129 192 223 297 519	73 150 199 263 309+C 315+C 489	
HB-170	M7b1a1	129 223 297 527	73 150 199 263 309+C 315+C 489	
HB-242	M7b1a1	129 192 223 297 304	73 150 199 204 207 263 309+C 315+C 489	
HB-275	M7b1a1	129 192 223 297	73 150 199 263 309+C 315+C 489	
HB-280	M7b1a1	129 192 223 297	73 150 199 263 309+C 315+C 489	
HB-264	M7b1a1a	129 189 223 248 297	73 150 195 199 204 207 263 309+C 315+C 489	
HB-186	M7b1a1b	223 297	73 150 199 204 263 315+C 489	
HB-308	M7b1a1b	223 297	73 150 199 204 207 263 309+C 315+C 489	
HB122	M7c	153 266 519	73 146A 199 263 315+C 489 523-524d	
HB-197	M7c	075 223 293T 519	73 146 152 199 263 309+C 315+C 489 523-524d	
HB-248	M7c	075 223 260 293T 295 519	73 146 152 199 263 309+C 315+C 489 523-524d	
HB-229	M7c1a	223 294 295 311 319 422 519	73 146 199 263 315+C 489 523-524d	

HB-19	M8	184 189 223 298 319 470 471 473	73 263 315+C 489 750	
HB-79	M8	183T 184 189d 223 298 311 319 390 468 470A 471A 473A	73 146 263 309+2C 315+C 489	
HB-309	M8a2a	051 184 189 223 298 319 471	73 152 263 309+2C 315+C 489	
HB114	M8a3a	093 129 134 184 223 298 319	73 263 315+C 489	
HB-55	M9	111 129 223 235 300 362 519	73 150 263 309+2C 315+C 489	-
HB-244	M9	223 304 344 362 381 519	73 263 309+C 315+C 352+C 489	-
HB-F012	M9	111 129 223 235 300 362 519	73 150 263 309+C 315+C 489 523-524d	-
HB-257	M9	223 304 344 362 381 519	73 263 315+C 489	-
HB-35	M9a’b	223 362	73 153 263 315+C 489	
HB100	M9a1a1c	129 223 234 291 316 362	73 153 263 309+C 315+C 489	
HB-254	M9a1a1c	223 234 291 316 362	73 152 153 263 309+C 315+C 489	
HB138	M9a1b1	158 223 234 362 519	73 150 152 153 263 315+C 489	
HB-162	M9a1b1	158 223 234 311 362 519	73 150 152 153 263 309+C 315+C 489	
HB-274	M9a1b1	158 223 234 362 519	73 150 152 153 263 315+C 489	
HB-312	N11a1	183C 189 223 355 519	73 195 263 309+C 315+C 523-524d	
HB-51	N9a	223 257A 261 292 519	73 150 263 315+C	
HB-215	N9a	223 257A 261 390 519	73 150 263 315+C 456	
HB-227	N9a	223 257A 261	73 150 263 309+C 315+C	
HB-243	N9a	223 257A 261 311	73 150 263 309+2C 315+C	
HB-252	N9a	223 257A 261 311 390	73 150 195 263 315+C 524+AC	
HB-46	N9a1	111 129 189 223 257A 261	73 150 263 309+2C 315+C	
HB-16	N9a4b	092 145 172 223 245 257A 261	73 150 263 309+C 315+C 524+2AC 750	
HB-238	N9a4b	092 145 172 223 245 257A 261	73 150 263 309+2C 315+C 524+2AC	
HB-295	N9a4b	092 145 172 223 245 257A 261 519	73 150 152 263 309+C 315+C 524+2AC	

HB135	P6	182C 183C 189 311 362 519	73 263 315+C 523-524d	
HB106	R*	051 182C 183C 189 311 519	73 263 309+C 315+C 352+C	
HB118	R*, U	093 291 304 311 368 519	73 146 195 238 248d 263 309+C 315+C	
HB125	R11b1b	182C 183C 189 311 390 519	73 185 189 195 234 263 315+C	
HB-36	R22	169 249 265C 288 291 304 519	73 152 199 263 315+C 329	
HB-1	R30a	264 266A 361 399 519	73 152 153 204 210 263 309+C 523-524d 709 750	
HB-17	R9	124 148 184 304 309 390 519	73 263 309+C 315+C 750	
HB-188	R9b1	093 192 304 309 390 519	73 152 263 309+C 315+C 523-524d	
HB117	R9b1a2a	145 192 243 304 309 390 519	73 183 263 309+C 315+C 523-524d	
HB-30	R9b1b	124 148 304 309 380 390 519	73 263 309+C 315+C 523-524d	
HB-258	R9b1b	124 148 304 309 390 519	73 263 309+2C 315+C	
HB-267	R9c1a	157 256 266 304 311 335	73 152 236 249d 263 315+C	
HB-157	R9c1b1	093 157 304	73 151 263 315+C 479	
HB105	U	182C 183C 189 192 234 266A 519	73 150 210 263 309+2C 315+C 523-524d	
HB-305	U	231 519	73 146 263 309+C 315+C	
HB124	U2	051 182C 183C 189 519	73 152 214 263 315+C 352+C	
HB-306	U5b2a1	179 182C 183C 189 209 319	73 150 263 315+C	
HB98	Y2	126 231 311 519	73 146 151 153 263 310 482 523-524d	
HB-261	Y2	126 231 311	73 151 195 263 315+C 482 523-524d	
HB-5	Z	185 223 260 298	73 152 249d 263 309+C 315+C 489 750	
HB-47	Z	185 223 260 298	73 152 249d 263 309+2C 315+C 489	
HB-58	Z	185 223 260 298 311	73 152 249d 263 315+C 489 523-524d	
HB109	Z	185 223 260 298 442	73 143 152 249d 263 309+C 315+C 489	
HB-174	Z	185 209 223 260 298	73 152 249d 263 309+C 315+C 489 513	
HB-293	Z	185 223 260 298	73 152 249d 263 309+C 315+C 489 513	

HB-172	Z3a	185 223 260 298	73 152 207 249d 263 309+C 315+C 489	
spontaneously recovered samples				
YN-35	A	093 126 223 234 278 290 319 519	73 152 235 263 315+C 523-524d	
YN-128	A	092 223 256 290 319 362	73 152 235 263 309+C 315+C	
YN-146	A	223 284 290 319 362	73 152 235 263 309+C 315+C 523-524d	
YN-210	A	223 290 319	73 152 235 263 315+C 318 523-524d	
YN-222	A	093 129 136 223 284 290 295 319 362 519	73 152 235 263 309+C 315+C 523-524d	
YN-249	A	223 290 319 362	73 152 235 263 315+C 523-524d	
YN-275	A	223 290 319 362	73 152 235 263 309+C 315+C	
YN-292	A	223 260 290 319	73 146 152 195 235 263 309+C 315+C 523-524d	
YN-66	A11	223 234 290 293C 319 519	73 152 235 263 309+C 315+C 523-524d	
YN-208	A11	223 234 290 293C 319 519	73 152 235 263 309+C 315+C 523-524d	
YN-168	A13	124 167 223 290 319 362	73 152 200 235 263 309+2C 315+C 523-524d	
YN-203	A13	124 223 290 319 362	73 152 200 235 263 309+C 315+C 523-524d	
YN-211	A13	129 223 290 319 362	73 152 200 204 235 263 309+C 315+C 523-524d	
YN-225	A13	093 124 223 290 293T 319 362	73 152 200 235 263 309+C 315+C 523-524d	
YN-234	A13	223 290 319 362	73 143 152 200 235 263 309+C 315+C 523-524d	
YN-23	A14	093 223 290 319 335 362	73 151 152 200 235 263 309+C 315+C 523-524d	
YN-105	A14	223 290 319 362	73 151 152 200 235 263 315+C 334 523-524d	
YN-231	A14	132 223 256 290 319 362 381	73 93 151 152 200 235 263 309+C 315+C 523-524d	
YN-88	A15	223 290 319 362	73 152 207 235 309+C 315+C 523-524d	
YN-157	A5a	187 223 290 319	73 150 235 263 315+C 523-524d	
YN-194	A5b1	126 235 290 319 519	73 235 263 309+C 315+C 523-524d	
YN-36	B	114A 189 311 519	73 146 196 263 309+C 315+C 523-524d	
YN-86	B	188 189 266G 519	73 200 210 263 310 523-524d	

YN-44	B4	182C 183C 189 217 234 261 319 519	73 195 263 315+C 523-524d	
YN-68	B4	147 183 189 217 235 519	73 263 309+2C 315+C 523-524d	
YN-113	B4	167 182C 183C 189 217 218 261 519	73 146 152 263 315+C 350C 523-524d	
YN-135	B4	093 182C 183C 189 217 261 519	73 146 204 263 309+C 315+C 523-524d	
YN-169	B4	093 182C 183C 189 217 519	73 146 263 315+C 523-524d	
YN-229	B4	086 183C 189 217 223 519	73 146 263 309+C 315+C	
YN-269	B4	182C 183C 189 217 261 519	73 263 309+C 315+C	
N153	B4a3	092 182C 183C 189 217 261 399 519	73 200 263 310	
YN-149	B4a4	092 182C 183C 189 217 261 299 519	73 193 263 309+2C 315+C 523-524d	
YN-185	B4b1	136 183C 189 217 519	73 263 315+C 499	
YN-17	B4b1a	136 179 183C 189 217 519	73 150 207 263 315+C 499	
YN-43	B4b1a2	136 171T 179 183C 189 217 519	73 207 263 309+2C 315+C 499	
YN-125	B4c1b	140 183C 189 217 274 519	73 150 263 309+C 315+C	
YN-123	B4c1b2	140 183C 189 274 335 519	73 150 263 315+C	
YN-122	B4c1b2c1	136 140 183C 189 217 249 274 280 291 292 335 519	73 150 263 315+C	
YN-16	B4c1b2c2	129 140 166 183C 189 217 274 335 519	73 150 263 282 309+C 315+C	
YN-27	B4c1b2c2	129 140 166 183C 189 217 274 335 519	73 150 263 315+C	
YN-120	B4c1b2c2	129 140 166 183C 189 217 274 335 519	73 150 263 309+2C 315+C	
YN-82	B4c1c1	183 189 217 258C 311 356	73 150 195 214 263 315+C	
YN-126	B4g	181C 182C 183C 189 213 217 261 292 519	73 263 308 310d 523-524d	
YN-93	B4k	093 182C 183C 189 217 261 357 519	73 263 286d 309+C 315+C 523-524d	
YN-226	B4k	093 182C 183C 189 217 261 357 519	73 263 309+C 315+C 523-524d	
YN-246	B4k	093 182C 183C 189 217 261 299 519	73 263 315+C 523-524d	
YN-143	B5	140 183C 189 243 355 519	73 263 309+2C 315+C 523-524d	
YN-155	B5	183C 189 217 234 519	73 263 309+C 315+C	

YN-26	B5a	140 183C 189 261 266A 519	73 210 263 309+C 315+C 523-524d	
YN-34	B5a	140 183C 189 266A 482 519	73 210 263 309+C 315+C 523-524d	
YN-51	B5a	111 140 183C 189 266A 465 519	73 146 210 263 315+C 523-524d	
YN-58	B5a	140 183C 189 266A 484 485 490 491	73 210 263 315+C 523-524d	
YN-102	B5a	140 183C 189 234 266A 519	73 210 263 309+2C 315+C 523-524d	
YN-117	B5a	140 183C 189 266A 362 519	73 210 263 309+2C 315+C 523-524d	
YN-177	B5a	140 183C 189 266A 311 482 519	73 210 263 309+C 315+C 523-524d	
YN-257	B5a	131 140 183C 189 266A 519	73 150 210 263 315+C 523-524d 573+4C	
YN-160	B5a1d	140 182C 183C 189 261 266A 519	73 152 210 263 309+2C 315+C 523-524d	
YN-144	B5a2a1a	140 187 189 256 266G 519	73 93 207 210 263 309+C 315+C 523-524d	
YN-290	B5a2a1a	140 187 189 256 266G 519	73 93 210 263 315+C 523-524d	
YN-179	B5b2b	111 140 183C 189 234 243 256 463 519	73 103 131 146 263 309+2C 315+C 523-524d	
YN-138	B5b2c	111 140 183C 189 234 243 298 463 519	73 103 131 263 309+2C 315+C 481 523-524d	
YN-287	B5b2c1	111 140 182C 183C 189 234 243 463 519	73 103 263 309+C 315+C 481 523-524d	
YN-230	B6a	093 124 179 182C 183C 189 342	73 150 263 309+C 315+C 523-524d	
YN-60	C	223 298 327	73 249d 263 309+C 315+C 489 523-524d	
YN-61	C	223 270 298 327 519	73 146 249d 263 315+C 489	
YN-79	C	223 249 298 327	73 249d 263 309+C 315+C 489	
YN-85	C	223 298 327 519	73 249d 263 309+C 315+C 460 489	
YN-116	C	223 249 298 327	73 249d 263 309+C 315+C 489	
YN-142	C	172 223 298 327 519 540	73 146 249d 263 309+2C 315+C 489	
YN-154	C	223 298 327 342 519	73 146 249d 263 309+C 315+C 489	
YN-175	C	223 298 327 519	73 249d 263 309+C 315+C 489	
YN-216	C	183C 189 223 298 327 519	73 234 249d 263 309+2C 315+C 489	
YN-218	C	093 129 223 298 327 519	73 152 249d 263 315+C 489	

YN-242	C	183C 189 223 298 319 327 519	73 249d 263 309+2C 315+C 489	
YN-97	C4a1a	093 129 223 298 327 519	73 195 249d 263 315+C 489	
YN-204	C4a2c	223 298 327 354 357 519	47 73 249d 263 309+C 315+C 489 523-524d	
YN-19	C4b8a	093 223 298 327 519	73 249d 263 309+C 315+C 466 489	
YN-159	C5	223 288 298 300 327 519	73 249d 263 309+C 315+C 489	
YN-29	D	182C 183C 189 223 274 362 519	73 263 298 310 316 489	5178A 5231 5423
YN-53	D	223 249 362	73 263 315+C 489	5178A
YN-83	D	172 223 362	73 263 309+C 315+C 489	5105 5178A
YN-127	D	223 362 519	73 263 315+C 489 523-524d	5178A
YN-132	D	223 249 293T 304 352 362	73 173 263 315+C 489	5178A
YN-170	D	093 192 223 271 316 362	73 184 263 315+C 489	5178A
YN-176	D	223 362 519	73 195 263 315+C 489 523-524d	5178A
YN-181	D	223 362	73 195 263 315+C 489	5178A
YN-186	D	223 362	73 263 309+C 315+C 489	5178A
YN-200	D	223 362	73 150 153 263 315+C 408A 489	5178A
YN-221	D	223 362 519	73 263 315+C 390 489 523-524d	5178A 5237
YN-247	D	223 360 362 519	73 263 315+C 390 489 523-524d	5178A
YN-255	D	223 249 362	73 263 309+C 315+C 489	5178A
YN-264	D	223 362 497 519	73 94 263 315+C 489	5178A
YN-289	D4	092 223 294 299 362 519	73 146 263 315+C 489	5178A
YN-198	D4a1b1	129 223 309 362 519	73 152 228 263 309+C 315+C 489	
YN-141	D4a3b2	129 223 249 311 362	73 152 263 315+C 466 489	
YN-129	D4b1a1	223 298 319 519	73 152 263 315+C 489	
YN-45	D4b2b	223 243 244 362 519	73 194 263 309+C 315+C 390 489 523-524d	
YN-268	D4b2b2b	172 182C 183C 189 362	73 150 207 263 309+2C 315+C 489 523-524d	

YN-62	D4e1a	092 187 223 362	73 94 204 207 263 315+C 489 508	
YN-95	D4e1a	092 223 362	73 94 204 207 263 309+C 315+C 455+T 489 508	
YN-288	D4e1a	092 223 319 362	73 94 263 315+C 489	
YN-94	D4g2	223 362 526	73 263 298 309+C 315+C 489	
YN-46	D4g2a	223 274 362	73 263 298 315+C 489	
YN-81	D4g2a	093 104 223 274 362	73 152 234 263 298 310 489	
YN-109	D4g2a	223 274 362	73 263 298 309+2C 315+C 489 513+CA	
YN-22	D4i	093 223 294 362	65 73 195 237 263 296 315+C 489 501	
YN-240	D4i	223 294 362	73 263 309+C 315+C 489	
YN-261	D4j1a2	086 223 271 362	73 263 309+C 315+C 489	
YN-101	D4j3	184 189 223 311 362	73 200 263 309+C 315+C 489	
YN-50	D4j8	140 174 223 362	73 263 309+C 315+C 489 523-524d	
YN-63	D4s	223 362 519	73 199 263 315+C 489	
YN-114	D5	092 182C 183C 189 223 266 362	73 150 214 263 315+C 489 523-524d	
YN-193	D5	182C 183C 189 223 261 266 362 519	73 150 263 309+2C 315+C 489 523-524d	
YN-108	D5a2	092 164 172 182C 183C 189 223 249 362	73 150 263 309+2C 315+C 489 523-524d	
YN-212	D5a2a	164 172 182C 183C 189 223 266 362	73 150 200 263 309+C 315+C 489 523-524d	
YN-156	D5a2a1	092 164 172 182C 183C 189 223 266 362	73 150 235 263 315+C 489 523-524d	
YN-163	D5b	182C 183C 189 223 362	73 150 263 315+C 456 489	
YN-188	D5b	183C 189 217 223 319 362 519	73 150 189 263 315+C 456 489	
YN-206	D5b	183C 189 223 362 519	73 150 204 263 309+C 315+C 456 489	
YN-39	D5b1a1	167 183C 189 223 362	73 150 204 263 309+2C 315+C 456 489	
YN-70	F	304	73 249d 263 315+C	
YN-133	F	304	73 249d 263 309+2C 315+C	
YN-227	F	182C 183C 189+C 304 519	73 249d 263 309+2C 315+C 523-524d	

YN-236	F	183C 189 304 519	73 249d 263 309+C 315+C 523-524d	
YN-293	F	092 126 183C 189 304 390 519	73 195 249d 263 309+2C 315+C 368 523-524d	
YN-01	F1	182C 183C 189 232A 249 304 519	73 152 249d 263 309+2C 315+C 523-524d	
YN-30	F1	182C 183C 189 304 519	73 153 249d 263 309+2C 315+C 523-524d	
YN-172	F1	183C 189 243 304 519	73 249d 263 309+C 315+C 523-524d	
YN-196	F1	189 274 304 519	73 208 249d 263 309+C 315+C 523-524d	
YN-282	F1	129 182C 183C 189 304 519	73 249d 263 309+2C 315+C 523-524d	
YN-14	F1a	129 172 304 519	73 249d 263 315+C 523-524d	
YN-33	F1a	129 172 304 519	73 185 189 207 249d 263 309+C 315+C 523-524d	
YN-136	F1a	129 172 304 519	73 249d 263 309+2C 315+C 521-524d	
YN-162	F1a	129 172 304 519	53 54 71d 73 152 249d 263 309+C 315+C 318 523-524d	
YN-184	F1a	129 172 304 362 519	73 249d 263 309+C 315+C 521-524d	
YN-244	F1a	129 172 304	73 249d 263 315+C 523-524d	
YN-52	F1a1	129 162 172 304 497 519	73 234 249d 263 315+C 523-524d	
YN-72	F1a1	129 162 172 243 304 519	73 249d 263 315+C 523-524d	
YN-119	F1a1	129 162 172 289 304 497 519	73 249d 263 309+C 315+C 523-524d	
YN-167	F1a1	129 162 172 189 304 519	73 94 249d 263 309+C 315+C 523-524d	
YN-228	F1a1	129 162 169 172 304 519	73 249d 263 309+C 315+C 523-524d	
YN-233	F1a1	129 162 172 519	09 73 249d 263 315+C 523-524d	
YN-283	F1a1	129 162 172 304 497 519	73 249d 263 315+C 523-524d	
N134	F1a1a	108 129 162 172 304 372A 519	73 186 249d 309+C 315+C 523-524d	
YN-25	F1a1a	108 129 162 172 304 519	73 249d 263 309+C 315+C 523-524d	
YN-137	F1a1a	108 129 162 172 304 519	73 150 249d 263 309+2C 315+C 523-524d	
YN-171	F1a1c	129 162 172 304 519	73 249d 263 315+C 523-524d 548	
YN-243	F1a1c	129 162 172 274 304 311 519	73 249d 263 309+C 315+C 523-524d 548	

YN-90	F1a1d	129 162 168 172 304 399 519	73 249d 263 309+2C 315+C 523-524d	
YN-124	F1a1d	129 162 172 304 399 519	73 152 249d 263 309+C 315+C 523-524d	
YN-64	F1a2	172 304 357 519	73 200 249d 263 309+C 315+C 523-524d	
YN-209	F1a2	093 162 172 304 497 519	73 249d 263 309+C 315+C 523-524d	
YN-54	F1a2a	172 304 465 519	73 249d 263 309+2C 315+C 521-524d	
YN-284	F1a2a	172 304 465 519	73 249d 263 309+C 315+C 521-524d	
YN-05	F1a3	129 172 304 519	73 249d 263 315+C 523-524d	
YN-12	F1a3	129 172 304 519	73 249d 263 315+C 523-524d	
YN-11	F1b	182C 183C 189 232A 249 304 311	73 249d 263 315+C 523-524d	
YN-55	F1b1	183C 189 232A 249 304 311	73 249d 263 315+C 523-524d	
YN-235	F1b1	183C 189 232A 249 304 311 519	73 146 204 207 249d 263 309+2C 315+C 523-524d	
YN-74	F1c1	129 182C 183C 189 299 304 357 519	73 152 249d 263 309+C 315+C 523-524d	
YN-76	F1c1a	111 129 304 519	73 152 234 249d 263 309+2C 315+C	
YN-87	F1c1a	111 129 304 519	73 152 234 249d 263 315+C	
YN-96	F1e1a	183C 189 194 195 304 355 519	73 249d 263 315+C	
YN-37	F2	185 266G 291 519	73 249d 263 315+C	
YN-214	F2a	203 256 291 304 519	73 249d 263 309+C 315+C	
YN-92	F2a1	203 304 325	73 183 249d 263 315+C	
YN-197	F2d		73 235 249d 263 315+C	
YN-254	F2d		73 228 235 249d 263 309+2C 315+C 574	
YN-56	F3a1	129 260 298 355 362	73 204 207 249d 263 309+C 315+C	
YN-06	F4a2	207 304 399	73 146 152 249d 263 281 309+C 315+C	
YN-252	F4a2	207 304 399	73 146 152 249d 263 281 309+C 315+C	
YN-118	G	093 184 223 290 362 519	73 247 263 315+C 489	5108
YN-165	G	185 189d 223 258C 278 362	73 263 309+C 315+C 489	5063 5108

YN-91	G1a1	223 304 325 362 519	73 150 263 309+C 315+C 489	
YN-98	G2a	221 223 227 278 304 362	73 263 309+C 315+C 489	
YN-278	G2a	093 223 224 227 278 362	73 263 309+C 315+C 489	
YN-201	G2a1d	183 186+T 189 223 278 362	73 260 263 315+C 489	
YN-107	G2a2	148 223 227 278 362 519	73 152 263 309+C 315+C 489	
YN-71	G2c	223 234 519	73 152 195 204 263 309+C 315+C 489	
YN-08	G3a	172 223 274 356 362	73 143 263 315+C 489 523-524d	
YN-166	I4a1	129 223 304 391 519	73 199 204 250 263 309+C 315+C 523-524d 573+5C	
YN-69	L3h1	223 274 311 519	73 146 263 309+C 315+C 489	
YN-110	L3h1	223 241 311 519	73 146 154 263 309+C 315+C 489	
YN-28	M*	223 311 519	73 94 150 263 309+C 315+C 489	
YN-140	M*	129 223 311	73 263 315+C 489 573+3C	
YN-148	M*	519	73 146 199 263 315+C 489 523-524d	
YN-178	M*	223	73 150 198 263 309+C 315+C 318 326 489	
YN-182	M*	066 223 311 398	73 263 315+C 489	
YN-195	M*	066 223 311	73 263 315+C 489	
YN-202	M10a1b	066 223 311 319	73 263 489 573+5C	
YN-89	M11	223 304	73 185 215 263 315+C 318 326 489	
YN-281	M11	172 223 519	73 215 263 309+C 315+C 318 326 471 489	
YN-259	M11b1a	172 223 286	73 146 198 215 263 315+C 318 326 489	
YN-189	M12a1	093 129 223 234 290 311	73 125 127 128 146 152 263 315+C 318 489	
YN-106	M12a1a1	058C 223 234 287 290 362	73 125 127 128 200 263 315+C 318 489 513 523-524d	
YN-65	M12a1b	148 172 189 223 234 290 519	73 125 127 128 146 195 263 315+C 489	
YN-77	M13a1b	145 148 188 189 223 362 381	73 152 263 309+C 315+C 489	
YN-134	M13a1b	145 148 188 189 223 381	73 152 263 315+C 489	

YN-31	M35b	223 362	73 199 263 315+C 489	
YN-180	M45	189 223 274 319 362	73 143 146 152 263 315+C 489	
YN-207	M46'61	129 223 270 362 519	73 152 263 309+C 315+C 489	
YN-272	M4a	145 176 223 261 311 519	73 263 315+C 489	
YN-84	M5	129 223 278 362	73 263 309+C 315+C 489	
YN-153	M61a	223 270 362 381 519	73 152 263 309+2C 315+C 489	
YN-258	M74a	093 223 311 362 381	63 64 66 73 215 263 315+C 489 523-524d	
YN-279	M74a	093 223 311 362 381	63 64 66 73 215 263 315+C 489 523-524d	
YN-164	M7b1	092 111 129 164 182C 183C 189 223 266 362	73 150 153 199 263 315+C 489 523-524d	
YN-192	M7b1	092 129 164 182C 183C 189 223 266 362	73 150 199 263 315+C 489 523-524d	
YN-42	M7b1a	178 189 223 297	73 150 199 204 263 309+2C 315+C 456 489	
YN-03	M7b1a1	129 192 223 266 297	73 150 159 199 263 315+C 489	
YN-100	M7b1a1	129 192 223 297	73 150 195 199 217 263 309+2C 315+C 332 489	
YN-152	M7b1a1	129 192 223 297 519	73 150 199 263 309+C 315+C 489	
YN-173	M7b1a1	129 223 297	73 150 199 263 309+C 315+C 489	
YN-224	M7b1a1	129 192 223 297	73 150 182 199 263 315+C 459d 489	
YN-232	M7b1a1	129 192 223 263 297	73 146 150 195 199 263 309+2C 315+C 332 489	
YN-251	M7b1a1	129 189 192 201 223 297 519	73 150 199 263 309+2C 315+C 489	
YN-262	M7b1a1	129 192 223 297	73 150 182 199 263 315+C 459d 489	
YN-80	M7b1a1a	129 189 223 297	73 150 199 263 489	
YN-215	M7b1a1a	129 189 223 248 297	73 150 199 204 207 263 315+C 489	
YN-217	M7b1a1a	129 189 223 248 293 297	73 150 199 204 207 263 315+C 489	
YN-223	M7b1a1a	129 189 223 248 297	73 150 199 204 207 263 309+C 315+C 489	
YN-267	M7b1a1a	129 189 192 223 297 519	73 150 199 263 309+C 315+C 489 513	
YN-219	M7b1a1a3	129 189 223 297	73 150 199 204 263 315+C 456 489	

YN-280	M7b1a1b	093 129 192 223 297 309	73 150 199 263 315+C 489	
YN-145	M7b1b	129 152 179 192 223 354 362	73 263 309+C 315+C 489	
YN-13	M7c1	223 293T 295	72 73 146 199 263 309+C 315+C 489 523-524d	
YN-150	M7c1	223 295 519	73 146 199 263 309+2C 315+C 489 523-524d	
YN-260	M7c1	075 193 223 293T 295 519	73 146 152 199 263 309+2C 315+C 489 523-524d	
YN-286	M7c1	223 295 519	73 146 152 199 263 309+C 315+C 489 523-524d	
YN-121	M7c1a1b	223 295 304 311 519	73 199 263 309+C 315+C 489 523-524d	
YN-190	M7c1c2	093 519	73 146A 199 204 263 309+C 315+C 489 523-524d	
YN-199	M7c1c2	362 519	73 146A 199 228 263 309+2C 315+C 489 523-524d	
YN-270	M8a	223 298 319	73 263 309+C 315+C 489 516	
YN-24	M8a2'3	184 223 298 311 319	73 204 263 309+C 315+C 489	
YN-112	M8a2'3	184 223 298 319 356	73 198 263 315+C 489	
YN-174	M8a2'3	184 209 223 298 311 319	73 263 309+C 315+C 489 523-524d	
YN-276	M8a2'3	184 209 223 298 311 319	73 263 309+C 315+C 489 523-524d	
YN-291	M8a3a	134 184 223 298 319	73 263 309+2C 315+C 489	
YN-274	M9a1a	223 234 316 362 526	73 153 263 315+C 489	
YN-115	M9a1a1	223 234 291 316 362	73 263 309+C 315+C 489 508	
YN-04	M9a1b1	092 158 223 234 274 311 362 519	73 150 152 153 263 315+C 489 513	
YN-241	M9a1b1	092 158 223 234 274 311 362 519	73 150 152 153 263 315+C 489 513	
YN-191	M9b	051 209 223 362 519	73 153 263 309+C 315+C 489 573+4C	
YN-250	N11a1	183C 189 223 355 519	73 151 195 263 309+C 315+C 523-524d	
YN-263	N11a1	145 183C 189 223 355 519	73 195 240 263 310 523-524d	
YN-104	N9a	183C 189 223 242 257A 261	73 150 151 263 309+2C 315+C	
YN-158	N9a	093 223 257A 261 519	73 150 263 309+C 315+C	
YN-103	N9a1	111 129 223 257A 261	73 150 263 315+C	

YN-271	N9a1'3	129 223 257A	73 150 263 309+C 315+C	
YN-130	N9a4	145 172 223 245 257A 261	73 150 263 268 315+C 524+2AC	
YN-67	N9b	182C 183C 189 223 234 519	73 263 309+2C 315+C	
YN-187	R11a	093 182C 183C 189 311 365 519	73 185 189 263 309+C 315+C	
YN-151	R11b1	182C 183C 189 311 390 399 519	73 185 189 263 309+C 315+C	
YN-07	R9b1	093 104 182C 183C 189 304 309 390 519	73 263 309+C 315+3C	
YN-183	R9b1	192 239 304 309 390 519	73 152 263 309+C 315+C 523-524d	
YN-238	R9b1	239 304 309 390 519	73 151 263 315+C	
YN-245	R9b1	192 239 304 309 390 519	73 152 263 309+C 315+C 523-524d	
YN-253	R9b1	169 304 309 390 519	73 263 309+2C 315+C	
YN-285	R9b1	209 278 304 309 390	73 263 309+C 315+2C 523-524d	
YN-57	R9b1a	192 304G 309 390 519	73 183 263 309+C 315+C 523-524d	
YN-21	R9b1b	124 148 290 304 309 390 519	73 263 309+2C 315+C	
YN-32	R9b1b	124 148 304 309 362 390 519	73 263 309+C 315+C	
YN-205	R9b1b	124 148 275 304 309 327 390 519	73 263 309+C 315+C 523-524d	
YN-213	R9b1b	093 124 148 170 304 309 390 519	73 263 309+C 315+C	
YN-99	R9c1a	157 256 304 335	73 249d 263 315+C	
YN-248	R9c1b1	093 157 304 519	73 151 263 315+C 479	
YN-75	U2	051 182C 183C 189 519	73 152 263 315+C 352+C	
YN-273	U5a1	192 256 270 399	73 263 315+C 524+AC	
YN-78	U5b	164 182C 183C 189 266 362	73 150 263 315+C 489 523-524d	
YN-111	U5b2a	129 182C 183C 189 352 355 519	73 150 152 185 189 263 309+C 315+C	
YN-73	W1	223 243 292 519	73 119 189 195 204 207 263 315+C	
YN-20	Z	136 185 223 260 274 298	73 152 249d 263 309+2C 315+C 489	
YN-131	Z	185 223 260 298 380	73 143 152 195 249d 263 309+C 315+C 489	

YN-161	Z	185 223 260 298 302	73 152 195 249d 263 309+C 315+C 489	
YN-277	Z	185 189d 223 224 260 261 298 302	73 152 153 185 188 200 249d 263 309+C 315+C 489	
YN-239	Z3a	185 223 260 298	73 152 204 207 249d 263 309+C 315+C 489	
YN-237	Z4	093 185 186 189 192 223 245 260 298	73 152 207 249d 263 309+2C 315+C 489	
YN-10	Z4a	185 223 260 264 298 302 317T 362	73 151 152 249d 263 309+2C 315+C 489	
healthy control samples				
N56	A	147 223 234 290 293 319	73 152 235 263 309+C 315+C 523-524d	
N83	A	223 248 290 319 362	73 152 182 235 263 309+2C 315+C	
N123	A	086 189 223 290 319 362	73 152 200 204 235 263	
N133	A	223 290 319 362	73 151 152 235 263 309+C	
N168	A	223 290 311 319 362	73 152 235 263 315+C 523-524d 543	
N177	A	223 274 290 319 362 519 527	73 152 235 263 309+C 315+C 523-524d	
N178	A	223 290 319 362	73 152 235 263 309+C 315+C 523-524d	
N248	A	223 284 290 319 362	73 152 235 263 309+C 315+C 523-524d	
N250	A	093 172 223 234 290 319 362	73 207 235 309+C 315+C 523-524d	
N296	A	223 290 319 362	73 151 152 235 248d 263 309+C 315+C 523-524d	
N301	A	223 234 290 293 304 319 519	73 152 235 263 309+C 315+C 523-524d	
N319	A	168 209 223 256 274 290 319 362 519 527	73 152 235 249d 263 315+C	
N335	A	168 209 223 256 274 290 319 362 519 527	73 152 235 263 309+C 315+C 523-524d	
N243	A11	223 234 290 293C 319 519	73 152 235 263 309+C 315+C 523-524d	
N329	A11b	223 234 290 293C 311 319 519 527	73 152 194 207 235 263 272 315+C 523-524d	
N12	A12	051 183C 189 223 290 311 319 362 519	73 152 234 235 248d 263 309+C 315+C 523-524d	
N30	A13	124 223 290 319 362	73 152 200 235 263 309+2C 315+C 523-524d	
N118	A13	223 290 319 362 519	73 195 200 235 263 309+C	
N173	A13	124 223 290 319 362	73 152 200 235 263 309+C 315+C 523-524d	

N289	A13	126 223 290 319 362	73 152 200 235 263 315+C 523-524d	
N29	A14	223 290 319 362	73 151 152 200 235 263 315+C 523-524d	
N65	A14	129 185 223 290 319 362	73 151 152 200 235 263 315+C 523-524d	
N93	A14	223 290 319 362	73 151 152 200 235 263 309+C 315+C	
N146	A14	223 290 319 362	73 151 152 200 235 263	
N273	A14	093 223 290 319 362	73 151 152 200 235 263 315+C 523-524d	
N108	A15	223 290 319 320 362	73 152 207 235 309+C 315+C	
N217	A15	223 274 290 319 362	73 151 152 204 207 235 309+C 315+C 523-524d	
N304	A15	093 169 223 290 319 356 362	73 146 152 207 235 309+C 315+C 523-524d	
N67	A15b	093 172 223 234 290 319	73 152 204 207 235 315+C 523-524d	
N188	A19	223 290 311 319 362 519	73 146 152 199 235 263 315+C 523-524d	
N97	A26	144 223 274 286 290 319 362	73 152 235 263 315+C 523-524d	
N236	A5b1	104 126 223 235 290 319 519	73 235 263 315+C 523-524d	
N27	B	189 234 243 463 519	73 103 131A 263 309+C 315+C 481 523-524d	
N139	B	188 189 266G 372 519	73 210 263 309+C 315+C 523-524d	
N147	B	266A 519	73 210 263 315+C 523-524d 547	
N182	B	183C 189 243 519	73 103 204 309+C 315+C 523-524d	
N02	B4	182C 183C 189 217 299 519	73 143 189 193 204 263 309+C 315+C 523-524d	
N31	B4	183d 186 189 217 519	73 152 200 263 309+C 316C 316+A	
N34	B4	108 182C 183C 189 217	73 263 309+2C 315+C 523-524d	
N43	B4	189 217 241 274 311	73 146 150 195 263 315+C	
N52	B4	129 182C 183C 189 217 519	73 263 309+2C 315+C 523-524d	
N62	B4	093 129 182C 183C 189 217	73 146 153 263 295 309+2C 315+C 523-524d	
N86	B4	182C 183C 189 217 261 299	73 193 263 309+2C 315+C 523-524d	
N111	B4	136 183C 189 217 260 519	73 263 309+C 315+C 499 523-524d	

N114	B4	189 189+C 217 234 357 519	20 26 73 263 309+2C 315+C	
N166	B4	129 182C 183C 189 217 261 354	73 263 309+2C 315+C 523-524d	
N179	B4	182C 183C 189 217 299 519	73 193 263 315+C 523-524d	
N181	B4	183C 189 215T 217 519	55 56 73 263 309+2C 315+C	
HB-N161	B4	183C 185C 189 217 519	73 152 263 309+C 316C 316+A	
N218	B4	182C 183C 189 217 249 261 357 519	41 73 263 309+2C 315+C 523-524d	
N251	B4	108 182C 183C 189 217 362G 519	73 263 315+C 502 523-524d	
N253	B4	172 180 183C 189 217 519	73 263 316C 316+A 524+AC	
N277	B4	093 183C 189 217 234 519	73 150 263 315+C	
N282	B4	129 182C 183C 189 217 519	73 263 309+2C 315+C	
N285	B4	129 182C 183C 189 217 519	73 263 309+2C 315+C 523-524d	
N305	B4	108 182C 183C 189 217 362 519	73 263 315+C 523-524d	
N126	B4a3	092 182C 183C 189 217 261 399 519	73 263 309+C 315+C 523-524d	
N95	B4b1	136 183C 189 217 218 239 248 519	73 263 315+C 499	
N230	B4b1	051 136 172 183C 189 217 218 519	73 263 309+C 315+C 499	
N245	B4b1a2c	136 182C 183C 189 217 311 519	73 189 207 263 309+C 315+C 499	
N299	B4g	181C 182C 183C 189 213 217 261 292 519	73 152 263 315+C 523-524d	
N175	B4g2	182C 183C 189 213 217 245 261 289 292 301 519	61A 62 73 263 310 319 523-524d	
N142	B4h1	129 182C 183C 189 261 319 519	73 263 309+2C 315+C 523-524d	
N53	B4k	093 182C 183C 189 217 261 357 519	73 263 315+C 523-524d	
N90	B4m	182C 183d 189 217 240 261	73 263 309+2C 315+C 523-524d	
N54	B5	140 183C 189 243 360 519	73 103 263 309+C 315+C 523-524d	
N88	B5	111 140 183C 189 463 519	73 204 207 263 309+2C 315+C 523-524d	
N242	B5	140 182C 183C 189 243 304 519	73 103 152 263 310 523-524d	
N294	B5	129 140 183C 189 243 300 519	73 103 152 210 263 315+CTC 523-524d	

N61	B5a	140 183C 189 266A 519	73 210 263 309+2C 315+C 523-524d	
N125	B5a	140 183C 189 266A 519	73 210 263 315+C 523-524d 589A	
N155	B5a	140 183C 189 222 266A 519	73 210 263 315+C 523-524d	
N162	B5a	140 183C 189 266A 519	73 210 263 315+C 523-524d	
N191	B5a	140 183C 189 266A 519	73 210 263 309+C 315+C 523-524d	
N223	B5a	140 183C 189 257 266A 519	73 210 263 309+C 315+C 523-524d	
N330	B5a	093 140 183C 189 266A 361 390 399 519	73 152 204 210 263 309+C 315+C 523-524d	
N334	B5a	140 183C 189 220 266A 519	73 210 263 309+C 315+C 523-524d	
N291	B5a1c1a1	140 183C 189 262 266A 519	64 73 210 263 315+C 523-524d	
HB-N86	B5a1d	140 182C 183C 189 261 266A 519	73 152 210 263 309+2C 315+C 523-524d	
N214	B5a1d	129 140 173 182C 183C 189 261 266A 519	73 152 210 263 309+2C 315+C 523-524d	
N41	B5a2	140 183C 189 266 519	73 210 263 309+2C 315+C 523-524d	
N73	B5a2	140 183C 189 266	73 195 210 263 309+C 315+C 523-524d	
N78	B5a2	140 183C 189 266 519	73 210 263 309+2C 315+C 523-524d	
N225	B5b4	140 183C 189 193+C 243 274 519	73 103 146 152 204 263 309+C 315+C 523-524d	
N131	B6a	093 179 182C 183C 189 298	73 150 263 309 310	
N01	C	183C 189 223 261 298 327 519	73 146 249d 263 309+C 315+C 489	
N21	C	223 298 327 519	45+C 73 249d 263 315+C 489	
N35	C	051 183C 189 223 278 298 327 519	73 249d 263 315+C 489	
N40	C	223 298 327 519	44+C 73 249d 263 315+C 489	
N55	C	124 223 298 311 327 519	73 146 249d 263 309+C 315+C 489	
N149	C	093 223 263 298 327 519	73 146 228T 249d 263 309+C 315+C 489	
N183	C	223 298 327 519	73 249d 263 309+2C 315+C 489	
N193	C	223 298 299 327 519	73 249d 263 315+C 489	
N260	C	182C 183C 189 223 239 298 327 519	73 249d 263 310 489	

N266	C	183C 189 223 261 298 327 519	73 249d 263 309+2C 315+C 489	
N339	C	223 292 298 327 519	73 146 151 249d 263 315+C 489	
N17	C4	223 298 319 327 519	73 249d 263 309+2C 315+C 489	
N85	C4	183C 189 223 298 311 327	73 249d 263 309+2C 315+C 489	
N135	C4	223 298 327 344 355 519	73 249d 263 309+C 315+C 489	
N151	C4	223 298 327 519	73 249d 263 315+C 489	
N290	C4a1	129 223 298 327 519	73 151 195 249d 263 315+C 489	
N163	C4a2a1	171 223 298 327 344 357 519	73 150 207 249d 263 315+C 489	
N300	C4a2c	124 223 298 327 354 357 519	47 73 249d 263 309+C 315+C 489 523-524d	
N293	C7a2	126 183C 189 223 298 327 519	73 249d 263 309+C 315+C 489	
N318	CZ	140 183C 189 223 260 298 519	73 152 249d 263 315+C 489	
N121	D	223 259 298 299 362	73 263 315+C 489	5178A
N23	D4	093 176 223 362	73 94 194 263 309+C 315+C 489	5178A
N36	D4	223 362	73 185 263 309+C 315+C 489	5178A
N145	D4	129 188+C 189+C 223 311 316 362 519	73 150 152 263 295A 310 489	5178A
N186	D4	184 223 311 362	73 263 315+C 489	5178A
N313	D4	185 189d 223 232A 319 362	73 263 315+C 489 523-524d	5178A
N25	D4	086 172 209 223 362 526T	73 150 263 315+C 489	5178A
N264	D4	114 223 294 311 362	73 146 263 315+C 489	4936 5178A
N307	D4	223 311 362	73 263 315+C 489	5178A
N20	D4a	129 162 223 362 519	73 152 263 309+C 315+C 489	
N71	D4a	129 223 362 519	73 152 194 263 309+C 315+C 489	
N137	D4a	129 223 305 362 519	73 152 263 315+C 489	
N192	D4a	129 223 362	73 152 263 309+C 315+C 489	
N270	D4a3b2	129 223 249 278 311 362	73 152 263 309+C 315+C 489	

N274	D4a3b2	129 223 249 311 362	73 152 263 315+C 489	
N50	D4b	223 311 362 519	73 150 194 263 315+C 489 523-524d	
N227	D4b2b	223 362 519	73 194 263 315+C 390 489 523-524d	
N337	D4b2b	223 311 362 519	73 150 194 263 315+C 489 523-524d	
N235	D4b2b2b	172 362 519	73 194 263 315+C 489 523-524d	
N315	D4e1a	092 223 362	73 94 263 315+C 489 523+C	
N256	D4g2a	223 274 362	73 146 263 298 309+2C 315+C 489	
N47	D4h1c	174 223 244 311 362	73 152 263 315+C 489	
N143	D4h3b	148 223 249 265C 301 342 362 519	73 152 263 309+2C 315+C 489	
N280	D4h4a	086 126 188+C 189+C 223 295 297 299 311 362	73 150 151 152 263 309+2C 315+C 489	
N150	D4j1a2	086 223 271 362	73 263 309+C 315+C 489	
N57	D4k	192 223 519	73 195 263 309+C 315+C 489	
N263	D4k	192 223	73 195 263 309+C 315+C 489	
N77	D5	051 189 223 362 390 519	73 146 150 151 152 263 309+C 315+C 489	
N100	D5	189 223 362 519	73 150 152 263 309+C 315+C 456 489	
N130	D5	172 189 223 362 519	73 150 152 263 309+C 315+C 456 489	
N42	D5a	092 172 182C 183C 189 362	73 150 263 315+C 489 523-524d 573+3C	
HB-N159	D5a2a	164 172 182C 183C 189 223 266 362	73 150 263 315+C 489 523-524d	
N232	D5a2a	164 172 182C 183C 189 223 266 362	73 150 200 263 309+C 315+C 489 523-524d	
N117	D5a2a1	092 164 172 182C 183C 189 223 243 266 362	73 150 263 315+C 489 523-524d	
N241	D5a2a1	092 164 182C 183d 186 189 223 266 362	73 150 263 309+C 315+C 489 523-524d	
N288	D5b1b2	182C 183C 189 223 357 362 519	73 150 185 263 309+2C 315+C 456 489	
N172	D5b1c1	092 148 183C 189 223 362 519	73 150 152 185 189 263 309+C 456 489 523-524d	
N38	D6c	183C 189 189+C 223 311 362	73 152 204 263 309+C 315+C 489 523-524d	
N26	F	182C 183C 189 304	73 249d 263 315+C 523-524d	

N28	F	183C 189 189+C 232 249 304	73 249d 263 315+C 523-524d	
N44	F	304	73 249d 263 316+C 318	
N45	F	182C 183C 189 304	73 249d 263 315+C 523-524d	
N76	F	172 183C 189 304	73 249d 263 309+C 315+C	
N82	F	126 183C 189 189+C 304 390	63 64 73 195 249d 263 523-524d	
N84	F	183C 189 243 304 519	73 185 249d 263 309+2C 315+C 523-524d	
N89	F	183C 189 243 304 519	73 185C 249d 263 309+2C 315+C	
N99	F	183C 189 304 519	73 249d 263 309+C	
N276	F	304	73 249d 263 315+C	
N09	F1	129 172 304	73 249d 263	
N11	F1	129 162 172 304 519	73 249d 263 309+C 315+C 523-524d	
N14	F1	183C 189 189+C 304 311 519	73 249d 263 309+C 523-524d	
N51	F1	129 183C 304 519	73 249d 263 315+C 523-524d	
N69	F1	129 183C 189 304 519	73 249d 263 309+C 315+C 523-524d	
N80	F1	108 129 162 172 189 304	10 26 73 150 249d 263 315+C 456	
N101	F1	129 172 304 519	73 146 249d 263 309+C 315+C	
HB-N68	F1	108 111 129 162 172 294 304 519	73 150 195 249d 263 315+C 523-524d	
N292	F1	183C 189 304 519	73 249d 263 309+C 315+C 523-524d	
N72	F1a	129 172 295 304 519	73 249d 263 315+C 523-524d	
N132	F1a	129 172 304 519	73 189 207 249d 263 309+C	
N169	F1a	162 172 304 519	73 249d 263 309+C 315+C 523-524d	
N312	F1a	129 172 304 519	73 249d 263 315+C 523-524d	
N324	F1a	129 172 304 519	73 249d 263 315+C 523-524d	
N298	F1a1	129 140 162 172 189 304 519	73 94 249d 263 309+C 315+C 523-524d	
N49	F1a1a	108 129 162 172 304	73 150 195 249d 263 315+C 523-524d	

N102	F1a1a	108 129 162 172 304 519	73 249d 263 297 315+C 523-524d	
N254	F1a1a	108 124 129 162 172 304 519	73 150 249d 263 309+C 315+C	
N325	F1a1a	108 129 162 172 292 304 519	73 152 195 249d 263 315+C 523-524d	
N19	F1a1c	129 162 172 304 519	73 249d 263 309+C 315+C 523-524d 548	
N171	F1a1c	129 162 172 304 519	73 249d 263 309+C 315+C 523-524d 548 549	
N287	F1a1c	129 162 172 304 519	73 249d 263 309+C 315+C 523-524d 548	
N154	F1a2a	172 295 304 465 519	73 249d 263 309+2C 315+C 521-524d	
N224	F1a3a	129 172 304 519	73 249d 263 315+C 523-524d	
N189	F1b	182C 183C 189 232A 249 304 311 344 519	73 152 249d 263 309+2C 315+C 523-524d	
N222	F1c1a1	111 129 266 304 519	73 152 195 249d 263 309+C 315+C 523-524d	
N212	F1e3	183C 189 241 300 304 311 519	73 150 195 198 249d 263 309+2C 523-524d	
N262	F2	185 266G 291 304 519	73 249d 263 309+2C 315+C	
N271	F2	145 185 266A 291 304 519	73 249d 263 315+C	
N58	F2a	203 212 291 304	73 249d 263 309+C 315+C	
HB-N77	F2a1	203 224 304 519	73 249d 263 315+C 523-524d	
HB-N158	F2a1	124 167 203 304 318 519	73 146 249d 263	
N70	F2b1	092A 291 304	73 249d 263 315+C 523-524d	
N75	F2h	304 519	73 195 249d 263 315+C	
N257	F2h	233 304	73 195 249d 263 310	
N267	F2i	221 304 519	73 152 195 249d 263 275 309+C 315+C	
N46	F4b	129 218 304 311	73 249d 263 315+C 573+5C	
N295	G	223 278 362	73 263 315+C 489	4991 5108
N336	G	223 362	73 263 309+C 315+C 489	5108
N16	G1a	093 168 177+T 192 223 271	73 150 184 189 263 315+C 489	
N249	G1a	223 292 362	73 150 239 263 309+C 315+C 376+C 489 524+AC 527	

N255	G1a1	223 325 362 519	73 150 195 263 315+C 489	
N343	G1a1	223 325 327 362 519	73 150 195 263 315+C 489	
N115	G2a	051 169 223 227 278 362 519	73 151 263 309+2C 315+C 489	
N216	G2a	223 227 278 362	73 263 309+C 315+C 489 516	
N59	G2a1h	189 223 227 234 278 311	73 263 315+C 455+TT 489	
N157	G2a2	223 227 278 362 519	73 152 263 309+C 315+C 489 523-524d	
N234	G2a2a	093 223 227 278 362	73 150 152 207 263 302+C 315+C 489 523-524d	
N03	G2c	223 362	73 152 195 235 263 309+2C 315+C 489	
N63	G3	189 223 265 274 362	73 143 152 263 315+C 489	
N309	G3	192 223 274 316 327 362	73 184 263 309+C 315+C 489	
N60	M	223 359 362	73 263 309+C 315+C 489	
N228	M	136 295 319 519	73 146 199 263 315+C 489 523-524d	
N37	M*	051 223 271 293	73 152 263 315+C 489	
N74	M*	092 140 183C 189 189+C 243	73 103 150 151 263 310 311 489	
N105	M*	066 223 311	73 263 309+C 315+C 489	
N124	M*	188+C 223 311 327A 519	73 263 309+C 315+C 417 489	
N138	M*	201 223 362	73 152 263 309+C 315+C 489	
N180	M*	126 223 362 519	73 263 315+C 489	
N185	M*	187 201 223 362	73 152 263 309+C 315+C 489	
N215	M*	223 362 519	73 198 200 204 263 309+C 315+C 318 326 489	
N233	M*	223 362	73 152 263 309+C 315+C 489	
N310	M*	223 234 260 292	73 146 263 315+C 489	
N314	M*	223 362 526	73 152 263 298 315+C 489 518	
N316	M*	129 223 297	73 150 263 309+C 315+C 489 523-524d	
N317	M*	185 223 260 298 311 519	73 194 263 315+C 489 523-524d	

N127	M10	093 129 193 223 311 357 497	73 146 263 309+C 315+C 489	
N128	M10	093 129 193 223 311 357 497	73 146 263 309+C 315+C 489	
N284	M12a1	223 234 287 290 362	73 125 127 128 263 309+C 315+C 318 489 523-524d	
N226	M13'46'61	223 362 519	73 152 199 263 279 315+C 489	
N340	M37	111 187 189 223 362 399 519	73 194 263 315+C 489 522d	
N306	M5	129 223 362	73 263 309+C 315+C 489 517	
HB-N61	M7	129 223 297	73 150 159 199 263 315+C 489	
N174	M7	189 223 288 295 519	73 146 152 188 199 263 309+C 315+C 489 523-524d	
N194	M7	129 183C 189 223 248 297	73 150 195 199 204 207 263 309+C 315+C 489	
N272	M7	086 297 324 399	73 199 263 315+C 489	
N322	M7	068 126 182C 183C 189 223 297	73 150 199 204 263 309+2C 315+C 456 489	
HB-N82	M71a1a	223 269 271 311	73 150 151 263 309+C 315+C 489	
N279	M71a1a	223 269 271 311	73 150 151 263 302+C 309+C 315+C 489	
N302	M71a1a	111 223 269 271 311	73 150 151 263 315+C 489	
N140	M71b	223 260 264 271 519	73 151 263 309+C 315+C 489	
N148	M71c	093 192 223 271 316 362	73 184 263 315+C 489	
N66	M74	223 278 311 362	63 64 66 73 146 263 309+C 315+C 489 574C	
N240	M74a	093 223 311 362 381	63 64 66 73 215 263 309+C 315+C 489 523-524d	
N338	M75	068 126 182C 183C 189 223 325 519	73 146 150 152 195 263 309+2C 315+C 489	
N08	M7a	183C 189 189+C 209 223 519	73 263 309+C 315+C 489	
N107	M7b	129 189 297 298 325	73 150 199 263 310 489	
N116	M7b	129 189 223 248 297	73 150 152 199 204 207 310 489	
HB-N89	M7b1a	129 223 297	73 150 199 204 263 271 309+C 315+C 489	
N259	M7b1a	129 192 223 297	73 150 182 199 263 315+C 459d 489	
N239	M7b1a1	129 223 297	73 150 159 199 263 309+C 315+C 489	

N244	M7b1a1	129 223 297	73 150 159 199 263 309+C 315+C 489	
N283	M7b1a1	129 192 223 297	73 150 199 263 315+C 489	
N297	M7b1a1	129 223 297	73 150 159 199 263 309+C 315+C 489	
N332	M7b1a1	129 223 297	73 150 159 199 263 315+C 489	
N286	M7b1a1a	093 129 183C 189 193+C 223 297	73 150 199 204 263 309+2C 315+C 489	
N303	M7b1a1a	129 189 223 297	73 150 199 204 207 263 315+C 489	
N159	M7b1a1a3	129 189 189+C 223 297	73 150 199 204 263 309+C 456 489	
N261	M7b1a1b	150 223 297	73 150 199 204 263 309+2C 315+C 489	
N281	M7b1a1b	223 297	73 150 199 204 263 309+C 315+C 489	
N331	M7c	223 239 293T 295 519	73 146 199 263 309+C 315+C 489 523-524d	
N91	M7c1	189 223 278 519	73 146 199 263 309+C 315+C 489 523-524d	
N252	M7c1	223 295 519	73 146 199 263 309+C 315+C 319 489 523-524d	
N18	M7c1c2	519	73 146A 199 263 315+C 489 523-524d	
N219	M7c2	172 223 311 519	73 146 152 263 315+C 489 523-524d	
N341	M7c2a	172 223 274 291 311 519	73 146 263 315+C 489 521d 523d	
N24	M8a	184 223 293 298 319	73 152 263 315+C 489 589A	
N110	M8a	184 189 223 298 319 470A 471A 473A	73 263 309+2C 315+C 489	
N187	M8a	134 223 298 319	73 263 309+C 315+C 489	
N342	M8a	184 189 223 298 311 319 390 468 470 471 473	73 146 263 309+C 315+C 489 523+C	
N48	M8a2a1	004 184 189 223 298 319	73 152 263 309+C 315+C 489	
N39	M9	362 519	73 153 183 263 309+C 315+C 489	
N92	M9	158 223 234	73 150 152 153 195 263 315+C 489	
N96	M9a	218A 223 234 316 362 465	73 146 263 309+2C 315+C 489	
N160	M9a1a	129 223 234 316 362	73 263 315+C 489	
N311	M9a1a	223 234 316 362	73 146 153 263 315+C 489	

HB-N88	M9a1a1c	093 223 234 291 316 362	73 153 263 309+C 315+C 489	
N103	M9a1b1	093 158 223 234 362 519	73 150 152 153 263 315+C 489	
N229	M9a1b1	158 179 183 223 234 362 519	73 150 151 152 153 263 310 489	
N119	M9a1b2	223 234 362 519	73 150 152 153 263 315+C 489	
N246	M9a'b	223 362	73 153 263 315+C 489	
N269	N11a1	189 223 355 519	73 195 198 263 315+C 523-524d	
N221	N21	182 193 223 519	73 150 195 263 309+C 315+C 337d	
N321	N9	223 311 362 519	73 150 207 263 309+C 315+C 524+2AC	
N184	N9a	193 223 257A 261	73 150 195 263 309+C 315+C	
N238	N9a	172 223 257A 261	73 150 263 315+C	
N265	N9a	183C 189 223 257A 261 311	73 150 263 315+C 385	
N326	N9a	129 223 257A 261	73 150 185 263 309+C 315+C 513	
N161	N9a1	111 129 223 257 261	73 150 263 309+C 315+C	
N190	N9a1	111 129 223 257A 261 265T 278	73 150 263 309+2C 315+C	
N275	N9a1	111 129 189 223 257A 261 354	73 150 263 309+2C 315+C	
N129	N9b	181C 182C 183C 189 223 261 292 519	73 263 309+2C 315+C 523-524d	
N64	R	086 304 311	73 146 195 207 315+3C 390 523-524d	
N141	R	231 519	73 146 263 309+C 315+C	
N165	R	147 182+C 189d 217 235 519	73 263 309+C 315+C	
N170	R	147 182+C 189d 217 235 519	73 263 309+C 315+C	
HB-N164	R*, B	094 182C 183C 189	73 263 315+C 368	
N237	R11a	182C 183C 189 311 365 519	73 185 189 194 263 309+2C 315+C	
N15	R11b1	189 311 390 399 519	73 146 185 189 194 263 309+2C 315+C	
N104	R11b1b	182C 183C 189 311 390 519	73 185 189 195 234 263	
N113	R9b1	193 288 304 309 390 519	73 143 146 204 263 523-524d	

N07	R9b2	304 362 519	73 263 315+C	
N98	R9b2	304 362 519	73 263 315+C 316+C	
N164	R9c1b1	093 157 304	73 146 151 263 309+C 315+C 479	
N213	U5b2a1	086 182C 183C 189 304 519	73 150 237 248d 263 309+C 315+C 523-524d	
N68	Y1	126 231 266 311 519	73 146 207 263 309+2C 315+C	
N247	Y1	126 231 249 266 319 399 519	73 146 263 309+2C 315+C	
N106	Z	185 189d 223 260 298 380	73 249d 263 309+C 315+C 489	
N156	Z	185 223 260 298 302	73 152 249d 263 315+C 489	
N167	Z	093 129 185 223 260 298	73 151 152 249d 263 315+C 489	
N176	Z	185 189d 223 260 298	73 152 249d 263 309+C 315+C 489	
N231	Z	185 223 260 263 298	73 152 249d 263 309+C 315+C 489 513	
N268	Z	185 223 260 294 298	73 152 249d 263 309+C 315+C 489 523-524d	
N308	Z	185 223 260 298	73 152 249d 263 309+C 315+C 489	
N333	Z	185 223 260 298 311 519	73 152 249d 263 309+C 315+C 489	
N220	Z4	185 223 260 298	73 151 152 249d 263 309+C 315+C 489	
N136	Z4a	185 223 260 298 302	73 151 152 249d 263 315+C 489	

Note: Positions are numbered according to the revised Cambridge reference sequence (rCRS);

[a] Suffixes “A”, “G”, “C”, and “T” indicate transversions; ‘‘d’’ indicates deletions, and ‘‘+’’ means insertion; indels are recorded at the last possible site.

[b] “-“means no mutation in region 4866-5461.

Supplementary Table S2. Recruitment criteria for chronic HBV infected (CHB) patients, spontaneously recovered (SR) subjects and healthy controls (HC).

CHB
HBsAg positive for at least 6 months ;
Anti-HCV and HCV RNA negative;
Anti-HDV and HDVAg negative;
SR
HBsAg negative
Anti-HBs and anti-HBc positive;
HBV-DNA negative;
Anti-HCV and HCV RNA negative;
Anti-HDV and HDVAg negative;
HC
HBsAg negative
Anti-HBs positive or negative
anti-HBc negative
Anti-HCV and HCV RNA negative;
Anti-HDV and HDVAg negative;

Supplementary Table S3. Published mtDNA data sets reanalyzed in this study.

abbreviation [a]	population	sample size	location	references
SH2	Han-Shanghai-2	120	Shanghai	[1]
TW	Han-Taiwan	155	Taiwan	[2]
HN1	Han-Hunan-1	82	Changsha, Hunan	[3]
SX1	Han-Shaanxi-1	85	Xi’an, Shaanxi	[3]
GD2	Han-Guangdong-2	69	Guangdong	[4]
LN1	Han-Liaoning	51	Fengcheng, Liaoning	[5]
SD1	Han-Qingdao	50	Qingdao, Shandong	[5]
XJ1	Han-Xinjiang	47	Yili, Xinjiang	[5]
GD1	Han-Guangdong_1	30	Zhanjiang, Guangdong	[5]
HB	Han-Wuhan	42	Wuhan, Hubei	[5]
YN1	Han-Yunnan	43	Kunming, Yunnan	[5]
SD2	Han-Shandong	76	Tai’an, Shandong	[6]
GS	Han-Gansu	45	Gansu	[7]
LN2	Han-Liaoning-2	51	Dalian, Liaoning	[7]
NM	Han-Neimeng	45	Chifeng, Neimeng	[7]
QH	Han- Qinghai	44	Xining, Qinghai	[7]
SX2	Han_Shaanxi_2	53	Xi'an, Shaanxi	[7]
AH	Han_Anhui	42	Hefei, Anhui	[7]
FJ	Han_Fujian	54	Changtin, Fujian	[7]
GX	Han_Guangxi	26	Tianlin, Guangxi	[7]
HN2	Han_Hunan_2	16	Changsha, Hunan	[7]
JS	Han_Jiangsu	67	Nanjing, Jiangsu	[7]
JX	Han_Jiangxi	23	Nanchang, Jiangxi	[7]
SH1	Han_Shanghai_1	56	Shanghai	[7]
SC1	Han_Sichuan	70	Weicheng, Sichuan	[7]
YN2	Han_Yunnan-2	59	Huize, Yunnan	[7]
ZJ	Han_Zhejiang	61	Hangzhou, Zhejiang	[7]
JL	Han-Jilin	51	Yan Bian, Jilin	[8]
Harbin	Han_Harbin	29	Harbin, Heilongjiang	[9]
MG	Han_Mongolia	17	Inner Mongolia	[9]
XJ2	Han_Xinjiang	29	Xinjiang	[9]
GD3	Han_Guangdong_2	102	Chaoshan	[10]
GD4	Han_Guangdong_3	170	Meizhou	[10]
YN3	Han-Yunnan	850	Yuxi, Yunnan	[11]
HN3	Han-Hunan	1005	Hunan	[12]
SC2	Han-Sichuan	435	Sichuan	[13]

[a] The abbreviations were correspond to Fig. 1.

References

1. Nishimaki, Y. *et al.* Sequence polymorphism in the mtDNA HV1 region in Japanese and Chinese. *Leg. Med. (Tokyo)* **1**, 238-249 (1999).
2. Tsai, L.C. *et al.* Sequence polymorphism of mitochondrial D-loop DNA in the Taiwanese Han population. *Forensic Sci. Int. Genet.* **119**, 239-247 (2001).
3. Oota, H. *et al.* Extreme mtDNA homogeneity in continental Asian populations. *Am. J. Phys. Anthropol.* **118**, 146-153 (2002).
4. Kivisild, T. *et al.* The emerging limbs and twigs of the East Asian mtDNA tree. *Mol. Biol. Evol.* **19**, 1737-1751 (2002).
5. Yao, Y.G., Kong, Q.P., Bandelt, H.J., Kivisild, T. & Zhang, Y.P. Phylogeographic differentiation of mitochondrial DNA in Han Chinese. *Am. J. Hum. Genet.* **70**, 635-651 (2002).
6. Yao, Y.G., Kong, Q.P., Man, X.Y., Bandelt, H.J. & Zhang, Y.P. Reconstructing the evolutionary history of China: a caveat about inferences drawn from ancient DNA. *Mol. Biol. Evol.* **20**, 214-219 (2003).
7. Wen, B. *et al.* Genetic evidence supports demic diffusion of Han culture. *Nature* **431**, 302-305 (2004).
8. Zhang, Y.J., Xu, Q.S., Zheng, Z.J., Lin, H.Y. & Lee, J.B. Haplotype diversity in mitochondrial DNA hypervariable region I, II and III in northeast China Han. *Forensic Sci. Int. Genet.* **149**, 267-269 (2005).
9. Powell, G.T., Yang, H., Tyler-Smith, C. & Xue, Y. The population history of the Xibe in northern China: a comparison of autosomal, mtDNA and Y-chromosomal analyses of migration and gene flow. *Forensic Sci. Int. Genet.* **1**, 115-119 (2007).
10. Wang, W.Z. *et al.* Tracing the origins of Hakka and Chaoshanese by mitochondrial DNA analysis. *Am. J. Phys. Anthropol.* **141**, 124-130 (2010).
11. Wang, D. *et al.* Mitochondrial DNA copy number, but not haplogroup, confers a genetic susceptibility to leprosy in Han Chinese from Southwest China. *PLoS One* **7**, e38848 (2012).
12. Zhang, W. *et al.* A matrilineal genetic legacy from the last glacial maximum confers susceptibility to schizophrenia in Han Chinese. *J. Genet. Genomics* **41**, 397-407 (2014).
13. Bi, R. *et al.* Mitochondrial DNA haplogroup B5 confers genetic susceptibility to Alzheimer's disease in Han Chinese. *Neurobiol. Aging* **36**, 1604 e1607-1616 (2015).
